# Parental beliefs, initiation of alcohol consumption, and binge drinking among Brazilian adolescents*

**DOI:** 10.1590/1518-8345.8036.4885

**Published:** 2026-10-16

**Authors:** Gyzelda Barros de Sousa, Patrícia Paiva de Oliveira Galvão, Juliana Yurgel Valente, André Ribeiro de Castro, Lidiane Nogueira Rebouças, Zila Van Der Meer Sanchez Dutenhefne, Samir Gabriel Vasconcelos Azevedo, Fabiane do Amaral Gubert

**Affiliations:** 1 Universidade Federal do Ceará, Departamento de Enfermagem, Fortaleza, CE, Brazil.; 2 Scholarship holder at the Coordenação de Aperfeiçoamento de Pessoal de Nível Superior (CAPES), Brazil.; 3 Universidade Federal de São Paulo, Departamento de Medicina Preventiva, Escola Paulista de Medicina, São Paulo, SP, Brazil.; 4 Scholarship holder at the Conselho Nacional de Desenvolvimento Científico e Tecnológico (CNPq), Brazil.; 5 Governo do Estado do Ceará, Secretaria Executiva Sobre Drogas do Ceará, Fortaleza, CE, Brazil.

**Keywords:** Health Promotion, Primary Prevention, School Health Promotion, Government Programs, Adolescent, Adolescent Health

## Abstract

**Objective:**

to analyze the association between adolescent alcohol consumption and parental beliefs.

**Method:**

a cross-sectional study nested within a cluster randomized controlled trial that evaluated the effectiveness of a program to strengthen family bonds in preventing drug use among adolescents. A total of 859 families with adolescents aged 10-14 years participated in the study. Participants completed a virtual questionnaire, and descriptive statistics and multivariate regression analyses were performed to assess the associations of interest.

**Results:**

parents who believed it was safer to teach their children to drink at home doubled the chances of their children starting to use alcohol (OR= 2.10). The belief that “if a teenager only drinks at parties, it will not affect their development” almost tripled (OR= 2.61) the chances of their children engaging in binge drinking. The study found that parents/guardians who used alcohol harmfully or were likely to be dependent increased the chances of their children consuming alcohol eightfold.

**Conclusion:**

the results suggest that more permissive parental beliefs about harmful alcohol consumption by parents can significantly increase the chances of early initiation and binge drinking in adolescence.

## Highlights

**(1)** Males predominate in alcohol use (56.3%) and binge drinking (63.3%).

**(2)** 12.3% have already started drinking alcohol, and 3.6% already engage in binge drinking.

**(3)** Drinking at home increases the chances of starting to drink alcohol by 1.97 times.

**(4)** Disapproval of drinking increases alcohol consumption by 2.37 times.

**(5)** Parents who drink harmfully increase the chances of alcohol consumption by 8.32 times.

## Introduction

In Brazil, approximately 1.5 million adolescents aged 13 or 14 have already tried alcohol, and of these, 13% have used illicit drugs at some point in their lives, with a higher prevalence among public school students^1^. Early alcohol use in adolescence may be related to the adaptations of this phase, which involve the incorporation of new habits and behaviors linked to the search for autonomy and decision-making^2^. In this context of change, adolescents are more exposed to behavioral risks, such as risky sexual behavior, violence, poor self-care, or reckless driving, when they begin using these substances^3^.

Adolescents’ exposure to these risks can be influenced by several factors, such as the lack of family structure, parental examples of substance use, and easy access to alcohol and other drugs^4^.

Parental alcohol consumption plays a key role in shaping adolescents’ habits and attitudes toward alcohol consumption. Thus, negative beliefs and attitudes and permissiveness in the presence of children are factors that contribute negatively to coping with alcohol in adolescence. Lack of parental support, parental alcohol consumption, and difficulty in setting limits are factors that can predispose adolescents to initiate or continue alcohol use^5^.

The relationship between parents’ beliefs and parenting practices is direct. These beliefs can be transmitted to children through communication mechanisms, such as narratives and instructions, and influence how children understand identities and the world around them^6^. Beliefs influence adolescents’ social-emotional skills and development. Thus, these beliefs shape how children deal with emotions and stress, a fundamental skill against alcohol^7^.

Parenting practices, by contrast, involve efforts aimed at adolescents’ well-being, learning, and the cultivation of values. These parenting practices in adolescent development indicate that more understanding, nonjudgmental, supportive, and counseling-oriented parenting styles are fundamental for adolescents^8^. Presumably, positive parenting practices are expected to contribute as protective factors for adolescents at risk for alcohol consumption.

It is important for nurses to understand the factors and beliefs that motivate adolescents to consume alcohol and engage in binge drinking or excessive drinking on certain occasions, as these professionals work to educate and promote health among families with adolescent children and in school environments, strengthening government actions such as prevention programs^9^. Demystifying inappropriate beliefs and investing in effective strategies against alcohol use and binge drinking is fundamental to improving adolescent health in Brazil, considering that it is a country with diverse cultures and ways of perceiving adolescents.

Given the above, evidence analyzing parental beliefs and alcohol consumption in adolescence is relevant, especially in Brazil, where the culture tends to be permissive regarding alcohol consumption. In this context, the family environment is conducive to understanding the context of alcohol use in adolescence. Thus, the objective was to analyze the association between alcohol consumption by adolescents aged 10 to 14 years^1^ and parental beliefs.

## Method

### Study design

This is a cross-sectional study, part of a cluster randomized clinical trial (RCT) designed to assess the effect of the government program to strengthen family ties, called Strong Families, on the prevention of alcohol and other drug use. The study analyzed baseline data from this RCT, collected prior to the intervention. The selection of cities and coordination were conducted during the program’s implementation, with supervision and monitoring by the now-defunct Ministry of Women, Family, and Human Rights (MMFDH) in 2021. The study was approved by the Research Ethics Committee of the Federal University of São Paulo, according to opinion 4,890,815.

The multicenter study was conducted in 12 Brazilian municipalities in the states of São Paulo, Rio de Janeiro, Mato Grosso, Paraíba, and Ceará. The Social Assistance Centers (CRAS, acronym in Portuguese) in these municipalities invited up to 15 families with at least one adolescent aged 10-14, as well as a responsible adult who could participate in the program meetings. The selected families were required to reside within 1 km of the CRAS. Families in which the adolescent was in the care of third parties (without any family ties), families with very high needs or challenges (such as drug addiction, family breakdown, and serious behavioral problems that could interfere with the group), and those involved in a program with the same objective or who had already participated in the Strong Families Program were not included.

CRAS were randomized from a single list of all CRAS in the municipalities participating in the study (in the process of agreeing to and signing an agreement with the federal government). Thus, the draw was not conducted by municipality but from a single list of CRAS across all municipalities, using Efron’s biased coin assumption to maintain a 1:1 ratio, in other words, the same number of controls CRAS and intervention CRAS. The lottery was conducted on the website www.random.org in two stages: 1) a lottery for CRAS and 2) a lottery of families within each CRAS. The list of eligible families was provided by the CRAS. Both lotteries were conducted by a researcher external to the main research team. The list of eligible families included a second list, designated the waiting list; thus, when a selected family declined to participate, the first family on the waiting list was invited.

The control group was recruited via a “program waiting list”, meaning that the control CRAS offered the intervention to participants only after the research was completed, i.e., one year after the start of the study. Thus, participants in the control group knew they would receive an intervention in the future but, at the time, were assigned to the control group and would receive the program only after data collection was completed. Thus, the final sample comprised 859 families.

The data was collected between November and December 2021. Participants responded to a virtual self-administered online questionnaire, made available on smartphones provided by the researchers, at the CRAS itself, in a private location and at a time scheduled in advance. Some collections were made at the families’ homes.

### Instruments and measures

The questionnaire was developed based on international instruments designed to evaluate the Strengthening Families Program (SFP 10-14)^10^, the program’s original name, as well as instruments that had been validated and previously used in studies evaluating drug prevention programs in Brazil^11^.

The program’s cultural adaptation was conducted by the University of Brasília during 2014 and 2015. All program materials were translated, with some words and cultural practices being adapted to the Brazilian context, such as replacing “rugby” and “basketball” with “soccer” and “family reunion” with “family time”. Visual adaptations were necessary, such as the “family crest” activity, which was transformed into “family flag”, requiring the production of new figures for the family flag.

Visual identity elements, such as images and icons, were adapted by Brazilian artists with characteristics reminiscent of children’s art, adapting colors and shapes, bringing the identity of the manuals closer to other programs implemented by the Federal Government, such as #Tamojunto and the Elos Program^12^. The materials were also reviewed, proposing changes to some written activities, which were then carried out orally, given the demand from participants with low literacy levels, considering the overload of facilitators in preparing the materials and the demand from participants to take the material home. Thus, permanent materials (cards and posters) and activity books for parents and young people were created in kit format. Even with these adaptations, the program content and the number of meetings remained unchanged with respect to the core elements of the program.

The outcome variable “alcohol use by adolescents” was analyzed using questions extracted from the World Health Organization (WHO) questionnaire used by the Brazilian Center for Information on Psychotropic Drugs (CEBRID, in Portuguese) in the VI Survey on Drug Use among Students^12^. For this study, the following questions were used to analyze alcohol use (yes or no) and lifetime binge drinking (yes or no): “Have you ever tried an alcoholic beverage?” and “Have you ever had 5 or more alcoholic drinks on a single occasion?”

As for “alcohol and other drug use by adults”, the Alcohol Use Disorders Identification Test (AUDIT) was applied, which screens people at risk of developing alcohol-related disorders^13^. The complete Brazilian version of the AUDIT^14^, which includes 10 items, covers three main areas: alcohol consumption (items 1 to 3), alcohol dependence (items 4 to 6), and negative consequences (items 7 to 10). The total score, which ranges from 0 to 40 points, is often calculated to assess the risk associated with alcohol use. This score allows participants to be classified into different categories, such as low-risk or abstinent alcohol consumption (0 to 7 points), risk of hazardous use (8 to 15 points), harmful alcohol use (16 to 19 points), or alcohol dependence (20 to 40 points)^15^.

The variable on parental beliefs was measured using five questions with dichotomous answers (yes or no): a) “I think it is safer to teach my child to drink at home while he or she is a teenager”, b) “If my child doesn’t drink, he will be excluded from his group of friends”, c) “Getting drunk as a teenager is just a phase and will soon pass”, d) “If a teenager only drinks at parties, it will not affect his development”, e) “Alcohol is not a drug, it is food”.

Sociodemographic data included gender, education level, age, and socioeconomic status, the latter collected using the Brazilian Association of Research Companies (ABEP, acronym in Portuguese) scale^16^. Higher scores indicate better economic status, and socioeconomic classes are classified from A (highest) to E (lowest), with the score generated from information on consumer goods and the education level of the head of the family. According to ABEP, Classes D and E can be grouped together. All variables used in this study were collected at the initial moment (baseline) and, therefore, were not randomized or influenced by the intervention.

The control and intervention groups were given the same virtual, audio-guided questionnaire at three points: before the start of the intervention, six months later, and 12 months later. The questionnaire was made available to participants via smartphones by researchers affiliated with the universities that promoted the study, in the absence of the program facilitator. Completion was anonymous, and the same questionnaire was applied at the three data collection points; however, some questions regarding the experience of participating in the program will be added to the follow-up questionnaires. The questionnaire was previously completed by field researchers, who provided a municipality and CRAS code. This code allowed for cluster (multilevel) analyses at two levels (municipality and service). The team’s previous experience with this confidential code model for time matching is noteworthy. A family and CRAS code was added to the individual code to allow matching between adolescents and identification of the CRAS cluster.

The data were collected using structured, virtual, anonymous, and self-administered questionnaires based on international references used for SFP 10-14 assessment^12^. The instrument was administered in a private location within the CRAS or in a facility designated by the service. The researchers provided tablets or computers to families to complete the questionnaires. When completing the questionnaires, parents and adolescents remained in separate locations to preserve confidentiality and privacy, particularly for adolescent participants, thereby avoiding the perception that their data would be shared with their parents.

Research involving multiple subjects and long follow-up periods tends to suffer from follow-up losses. Given the size of the controlled trial, multiple data imputation was employed to reduce bias and enhance the reliability of the results. Throughout the study, motivational and behavioral techniques were used with families, particularly adolescents, to encourage engagement in the program and completion of questionnaires.

Given the sensitivity of the topics addressed, some initiatives were adopted to ensure a reduction in information bias. The field procedures were: 1) informing the subjects that they had received a questionnaire on health and behaviors; 2) one or two trained researchers were present during the questionnaire completion process, and the presence of any CRAS professional was not allowed at this point of data collection; 3) the questionnaires were completed electronically on individual smartphones to avoid contact between researchers and the completed questionnaires, thus ensuring greater confidentiality of information; 4) field researchers emphasized more than once that the questionnaire was anonymous and that the data had not been and would not be accessed by anyone other than the researchers; 5) Young people answered the questionnaires in separate rooms, accompanied by a member of the research team.

### Statistical analysis

The variables studied were initially described by their measurement characteristics and summarized using absolute counts and prevalence estimates. Next, a hypothesis test was performed to assess the association between the variables of interest and alcohol use among adolescents using the chi-square test. Subsequently, the initial multivariate logistic regression model was considered, including explanatory variables with p-values ≤ 0.20 in the univariate regression. Next, a backward procedure was used to remove explanatory variables with p > 0.05, yielding a final model for each response variable, including the corresponding odds ratios (ORs), 95% confidence intervals (CIs), and p-values. The significance level adopted was 5%.

The analyses were performed on two samples: “*intention to treat*” (ITT), including all subjects who were randomized to participate in the study, according to the original allocation group, regardless of missing data; and “Complete Cases” (CC), in which only observations with complete data at baseline and follow-ups were considered; therefore, in this sample, cases with missing values were excluded.

Association measures were analyzed using Generalized Linear Mixed Models (GLMMs), which estimate the degree of dependence among data. Considering the hierarchical structure of the data, multilevel models were used to simultaneously verify differences in consumption prevalence between times and between groups, with weighting scales as modeling levels^12^. In terms of construction, organization, and methodological presentation, the study is aligned with the STROBE (Strengthening the Reporting of Observational Studies in Epidemiology) checklist.

## Results

Table 1 shows that the average age of adolescents was 12.6 years and that of parents was 39.5 years, with the majority (73.2%) classified in socioeconomic status categories D/E. Regarding lifetime drug use, 12.3% of adolescents reported consuming alcohol, and 3.6% engaged in binge drinking. As for the parents, the majority (96.3%) reported low-risk alcohol consumption. Regarding parental beliefs about adolescent alcohol use, it is noteworthy that 10.5% believed it was safer to teach their children to drink at home, and 20.8% stated that adolescents who only drink at parties will not have their development affected.

**Table 1:** Sociodemographic characteristics of adolescents and adults participating in the study (n = 859). Fortaleza, CE, Brazil, 2021

Variables	Adolescents		Adults	
	(n= 884)		(n= 884)	
	n*	%^†^	n*	%^†^
**Gender**				
Male	414	48.2	72	8.4
Female	439	51.1	782	91.0
Others	6	0.7	5	0.6
**Age (mean SD)** ^‡^	12.6±1.24		39.50±8.15	
**Socioeconomic status**				
A	---	---	8	0.9
B	---	---	33	3.8
C	---	---	190	22.1
D/E	---	---	628	73.2
**Lifetime drug use (adolescents)**				
Alcohol	104	12.3	---	---
Binge drinking	30	3.6	---	---
**Alcohol consumption by guardians**				
Low risk or abstainers	---	---	827	96.3
Risk	---	---	21	2.4
Harmful use or probable dependence	---	---	11	1.3
**Beliefs about drug use**				
Safe Drinking at Home^§^	---	---	89	10.5
Be Excluded^||^	---	---	149	17.5
Drunk in Adolescence^¶^	---	---	116	13.7
Drinking at Parties**	---	---	177	20.8
Alcohol is not a Drug^††^	---	---	45	5.3

*n = Sample size; ^†^Percentage value; ^‡^Average age value; ^§^I think it is safer to teach my child to drink at home while he is a teenager; ^||^If my child does not drink, he will be excluded from his group of friends; ^¶^Getting drunk as a teenager is just a phase and will soon pass; **If a teenager only drinks at parties, it will not affect their development; ^††^Alcohol is not a drug, it is food

Table 2 shows that alcohol use, as well as binge drinking, is more prevalent among male adolescents. Similarly, age is a relevant parameter, and it is evident that 13- and 14-year-olds consume more alcohol than 11- and 12-year-olds; this has been statistically proven. In the analysis of parental beliefs, for adolescents who consumed alcoholic beverages, 19.3% had parents who believed it was safer to teach their child to drink at home while they were adolescents (p=0.038). The beliefs that “getting drunk in adolescence is just a phase and will soon pass” and “if a teenager only drinks at parties, it will not affect their development” were associated with binge drinking by adolescents (p=0.037 and p=0.010, respectively).

**Table 2:** Distribution of alcohol use and binge drinking among adolescents, according to sociodemographic variables, parental beliefs, and parental alcohol consumption. Fortaleza, CE, Brazil, 2021

	Alcohol use in life					Binge drinking in life				
Variables	No		Yes		P^‡^	No		Yes		P^‡^
	n*	%^†^	n*	%^†^		n*	%^†^	n*	%^†^	
**Adolescent gender**										
Male	352	47.3	58	55.8	0.238	382	47.3	19	63.3	0.212
Female	388	52.1	45	43.3		420	52.0	11	36.7	
Others	5	0.6	1	0.9		6	0.7	0	-	
**Adolescent’s age**										
10-12 years	365	49	20	19.2	**<0.001**	370	45.8	9	30	0.088
13-14 years	380	51	84	80.8		438	54.2	21	70	
**Brazilian Association of Research Companies**										
A	7	0.9	0	-	0.684	8	1	0	-	0.813
B	28	3.8	5	4.8		31	3.8	1	3.3	
C	163	21.9	25	24		181	22.4	5	16.7	
D/E	547	73.4	74	71.2		588	72.8	24	80	
**Parents’ beliefs**										
Safe Drinking at Home^§^	71	80.7	17	19.3	**0.038**	84	97.7	2	2.3	0.603
Be Excluded^||^	129	87.2	19	12.8	0.771	141	95.9	6	4.1	0.669
Drunk in Adolescence^¶^	97	83.6	19	16.4	0.159	106	93	8	7.0	**0.037**
Drinking at Parties**	152	86.9	23	13.1	0.716	163	93.1	12	6.9	**0.010**
Alcohol is not a Drug^††^	37	86.1	6	13.9	0.741	42	95.5	2	4.5	0.731
**Alcohol consumption by parents**										
Low-risk consumption or abstinence	720	88.1	97	11.9	**<0.001**	778	96.4	29	3.6	0.769
Risky consumption	20	95.2	1	4.8		19	95	1	5.0	
Harmful use or probable dependence	5	45.5	6	54.5		11	100	0	-	

*n = Sample size; ^†^Percentage value; ^‡^p = p value at the 5% level (p ≤ 0.05); ^§^I think it is safer to teach my child to drink at home while he is a teenager; ^||^If my child does not drink, he will be excluded from his group of friends; ^¶^Getting drunk as a teenager is just a phase and will soon pass; **If a teenager only drinks at parties, it will not affect their development; ^††^Alcohol is not a drug, it is food

Table 3 highlights the association between parental alcohol consumption and beliefs regarding alcohol consumption and binge drinking by their children. It was observed that parents who “think it is safer to teach their children to drink at home” doubled the chances of their children starting to drink alcohol (OR= 2.10). Parents who reported believing that “if a teenager only drinks at parties, it will not affect their development” almost tripled (OR= 2.61) the chances of their children engaging in binge drinking. Another important finding was that parents/guardians who used alcohol harmfully or were likely to be dependent on alcohol increased the chances of their children consuming alcohol eightfold (OR= 8.91).

**Table 3:** Association between initiation of alcohol use, binge drinking (adolescents), parental beliefs, and parental alcohol consumption, according to OR* estimates and 95% confidence intervals**†** (N = 854). Fortaleza, CE, Brazil, 2021

	Univariate Logistic Regression						Multivariate Logistic Regression					
	Alcohol use in life			Practice of Binge drinking			Alcohol use in life			Practice of Binge drinking		
Variables	OR*	95%IC^†^	p^‡^	OR*	95%IC^†^	p^‡^	OR*	95%IC^†^	p^‡^	OR*	95%IC^†^	p^‡^
**Parents’ beliefs**												
Safe Drinking at Home^§^	1.82	[1.03;3.24]	0.041	0.68	[0.16;2.93]	0.605	2.10	[1.04;3.53]	0.036	---	---	--
Be Excluded^||^	1.08	[0.63;1.85]	0.771	1.22	[0.49;3.05]	0.669	---	---	--	---	---	--
Drunk in Adolescence^¶^	1.47	[0.86;2.53]	0.161	2.37	[1.03;5.47]	0.042	---	---	---	---	---	--
Drinking at Parties**	1.10	[0.67;1.80]	0.716	2.61	[1.23;5.53]	0.012	---	---	---	2.61	[1.23;5.53]	0.012
Alcohol is not a Drug^††^	1.16	[0.48;2.82]	0.741	1.29	[0.30;5.61]	0.732	---	---	--	---	---	--
**Alcohol consumption by parents**												
Low-risk consumption or abstinence	ref			ref			ref			ref		
Risky consumption	0.37	[0.05; 2.80]	0.336	1.41	[0.18; 10.91]	0.741	---	---	---	---	---	---
Harmful use or probable dependence	8.91	[0.67; 29.74]	<0.001	---	---	---	6.41	[1.84;22.34]	0.004	---	---	---

*OR = Odds Ratio; ^†^95% Confidence Interval; **‡**p = p value at the 5% level (p ≤ 0.05); ^§^I think it is safer to teach my child to drink at home while he is a teenager; ^||^If my child does not drink, he will be excluded from his group of friends; ^¶^Getting drunk as a teenager is just a phase and will soon pass; **If a teenager only drinks at parties, it will not affect their development; ^††^Alcohol is not a drug, it is food

## Discussion

The study identified that certain beliefs, such as teaching children to drink at home and thinking that drinking at parties is not a problem, are associated with early onset of alcohol consumption and binge drinking in adolescence. In addition, it was found that parents who engaged in harmful alcohol use or were likely to be dependent increased the chances of adolescents starting to drink alcohol by up to eight times, compared to those who did not engage in harmful use. The relevance of these data reiterates the need for nurses to work with parents and adolescents on beliefs.

The adolescents in this study had an average age of 12.6 years and consumption in about 12.3% of participants. This reality is very similar to the national context, as the average age is around 13 years^17^. When compared to countries with similar socioeconomic profiles, such as Mexico and Thailand, it is even more common for adolescents to experiment with alcohol at an early age, with a prevalence of 18% to 60% for this same age group^18^.

Regarding adults who consume alcohol, in this study, 5.8% used alcohol harmfully or were likely dependent on alcohol consumption, but it is worth noting that, in this sample, 91% of adults were women. Socially, women are more vulnerable, and in terms of alcohol consumption, this reveals a worrying scenario with consequences, emphatically, on mental health. Despite this, the Brazilian scenario is characterized in the opposite way, as it is noted that, among heavy drinkers, the prevalence was 3.2% among men and 0.6% among women^19^.

This study found a strong association between parental alcohol consumption and adolescent alcohol consumption. It was found that parents/guardians who used alcohol harmfully or were likely to be dependent on alcohol increased the chances of adolescent alcohol consumption by more than eight times. Similarly, a study in southern Brazil involving 1,170 adolescents showed that 48% of those who consumed alcohol reported that their parents drank alcohol and that 10.5% of these parents had had some problem with alcohol in the previous 30 days^20^. Another study in Brazil showed that alcohol consumption in families where both parents drank was associated with binge drinking among adolescents^21^. This correlation in young people’s consumption can be explained by the parental relationship and example set in the family; thus, young people mirror the behavior of the upbringing model to which they are subjected^22^.

Regarding parental beliefs about their children’s alcohol consumption, it was found that adults who responded that they considered it safer to teach their children to drink at home considerably increased the chances of their children starting to drink at an early age, with a 1.97 times greater likelihood of starting to drink. In addition, 20.8% of adults believed that if adolescents drank only at parties, this practice would not affect their development. This belief, in the context of the study, can almost double the chances of children engaging in binge drinking. Drinking at parties was identified as a trigger for excessive consumption, and the party environment was indicated as being associated with the place of first use of alcohol or other drugs. Binge drinking negatively influences adolescents’ learning processes and predisposes them to drug addiction, cigarette and illicit drug use, and withdrawal crises^23^.

In families where parents use psychoactive substances, there is a higher probability of consumption among their children. It is assumed that the significant association between parents/guardians who drink and polyconsumption among schoolchildren may reflect parental influence on adolescents^24^. The family environment is the adolescent’s main social group during the growth process and can act as a trigger for psychoactive substance use. The relationship between the behavior of young people and that of their parents may be associated with the reproduction of patterns observed in family life.

Parents/guardians who responded positively that “if a teenager only drinks at parties, it will not affect their development” resulted in a 2.61-fold increase in the chances of adolescents initiating alcohol use. Meanwhile, those who responded that “getting drunk in adolescence is just a phase and will soon pass” saw a 2.37-fold increase in the chance of alcohol consumption. Like these findings, a systematic review pointed out that less restrictive parental attitudes toward alcohol consumption among young people were associated with higher rates of initiation of alcohol use (OR) = 1.45, frequency of consumption (OR = 1.52), and episodes of drunkenness (OR = 1.58)^25^.

Parental behavior regarding alcohol use is linked to factors such as family environment, parenting style, boundaries, and cultural influences. Parents demonstrate awareness of the risks of alcohol and disapprove of its use by young people, but recognize its inevitable presence in culture. Thus, many adopt a balanced, reluctantly receptive stance, seeking to reconcile personal values with the promotion of their children’s autonomy and adaptation to social norms^26^.

The program showed a significant increase in parents’ ability to establish clear rules regarding their children’s drug use after 24 months of follow-up. The literature on substance use prevention consistently shows that well-defined and firm parental rules are an important protective factor against the onset and progression of substance use among adolescents. These rules help to create objective boundaries, allowing young people to understand family and social expectations regarding drug use, as well as reinforcing behavioral norms that discourage such use. Given this scenario, the program’s effects on reducing parental neglect and strengthening rule-setting practices can have a substantial impact on substance use prevention. Future research should investigate, through mediation models, whether these positive changes in parental behaviors, together with reduced neglect, directly influence the prevention of drug use among adolescents^27^.

It is worth noting that parental permissiveness regarding adolescent alcohol use is associated with long-term harm, regardless of the age at which consumption begins^28^. Given this, nurses play a strategic role in prevention, working to identify and deconstruct beliefs that normalize early alcohol consumption. It is essential that nurses develop planned educational interventions, particularly in school settings, to raise awareness among parents, students, and the community about the associated risks. These interventions help reduce risk factors and promote protective environments for adolescents.

Based on the above, when identifying the consumption profile among adolescents, it is important to consider that alcohol use can encourage violent behavior and fights, causing disinhibition and making adolescents more vulnerable^29^. Therefore, understanding the context of parental beliefs is relevant to health care, mainly because each parenting style has significant implications for children’s development and can influence their emotional, social, and cognitive well-being throughout their lives.

Interventions targeting parents that strengthen monitoring, effective communication, and emotional support have proven effective. These strategies directly reduce the availability and accessibility of alcohol to adolescents. In addition, they act indirectly, influencing adult behaviors and perceptions by promoting stricter regulatory environments and healthier parenting norms, both of which are associated with reduced alcohol consumption by minors^30^ ^)-(^ ^31^.

It is in this context that evidence-based prevention programs, such as the Strong Families Program proposed by the Brazilian Federal Government, propose interventions that address the quality of family relationships, and foster secure attachment between children and parents, including affective discipline, supervision, and monitoring by parents. The development of these skills occurs in a programmed, planned, and systematic manner, encouraging families to learn and practice these skills interactively, which is the most effective way of learning^32^.

Evidence indicates that parents tend to report higher levels of parental monitoring than their children, and that these discrepancies are greater for rule-related behaviors than for parental requests. In addition, over time, both parents and young people report a decrease in parental rules, although only parents report a reduction in requests. Given this, it is essential to examine young people’s perceptions of parental monitoring in relation to alcohol consumption, especially among adolescents with different patterns of use (i.e., abstainers and consumers), particularly when they reach the legal drinking age. In addition, specific rules on alcohol consumption have been shown to be effective in reducing use in intervention studies^33^ ^)-(^ ^34^.

Among the study’s limitations, it was conducted in only 12 Brazilian cities, which warrants caution when generalizing the results to the Brazilian population, given the country’s wide geographical and cultural diversity. In addition, the questionnaires were administered within a social support network, which may have influenced responses due to peer presence and social context. Although data collection was conducted separately from parents at certain stages, adolescents may have felt some degree of judgment or recrimination, even if only in their imagination.

The results provide important insights for professionals working in adolescent health, underscoring parental beliefs as a relevant public health issue. It is therefore essential to promote early interventions to prevent risky behaviors. In the Brazilian context, the permissive culture regarding alcohol consumption in adolescence, combined with weak control over the sale of alcoholic beverages, reinforces the urgent need for investment in research that deepens the understanding of these vulnerabilities.

## Conclusion

The study pointed out that permissive parental beliefs, such as consuming alcoholic beverages at home and at parties and harmful alcohol use by parents, can considerably increase the chances of early initiation of alcohol use by children and binge drinking. Thus, alcohol consumption by parents is an aggravating factor for adolescent health, causing damage to their lifestyle practices.

Thus, it is important to develop prevention programs for families with members still in early adolescence, which not only clarify the consequences of early alcohol use, but also work on parental education practices, modifying parental beliefs considered risky for alcohol use in adolescence, leading to prevention. Finally, there are several possibilities for nurses to work on protective factors by including beliefs about alcohol use and binge drinking with parents and adolescents within the scope of prevention. In addition, studies that assess parents’ beliefs and knowledge about the harm that alcohol can cause in the lives of adolescents are important to guide the development of these programs.

## Data Availability

Datasets related to this article will be available upon request to the corresponding author.

## References

[1] Instituto Brasileiro de Geografia e Estatística (2020). Pesquisa Nacional de Saúde Escolar 2019.

[2] Almeida CS, Abreu MNS, Andrade SN, Lana FCF (2021). Factors associated to alcohol use by adolescents. Texto Contexto Enferm.

[3] Silva DMR, Costa DT, Rocha GSA, Monteiro EMLM, Gomes BMR, Souza CFQ (2021). Association between family dynamics and use of alcohol, tobacco, and other drugs by adolescents. Rev Bras Enferm.

[4] Samudio Domínguez GC, Ortiz Cuquejo LM, Soto Meza MA, Samudio Genes CR (2021). Factors associated with consumption of illicit drugs in an adolescent population: A survey in low-income urban areas. Pediatr (Asunción) [Internet].

[5] Santos MHS, Valente JY, Gubert FA, Moreira HC, Sanchez ZM (2025). Parental practices as mediators of the effect of a family-based program on the prevention of substance use among Brazilian adolescentes. Child Youth Serv Rev.

[6] Melo PT, Santana SM (2020). Crack consumer: family beliefs influence in treatment. Pesqui Prát Psicossociais [Internet].

[7] List JA, Pernaudet J, Suskind DL (2021). Shifting parental beliefs about child development to foster parental investments and improve school readiness outcomes. Nat Commun.

[8] Arslan G (2024). Positive parenting, prosocial behavior, meaning in life, and subjective well-being in adolescents: a parallel and serial mediation analysis. Child Indic Res.

[9] Rocha RP, Galvão PPO, Sanchez ZM, Rebouças LN, Castro AR, Santos LES (2022). Body dissatisfaction, drug use, and associated factors among adolescents in three Brazilian cities. Rev. Latino-Am. Enfermagem.

[10] Allen D, Coombes L, Foxcroft DR (2007). Cultural accommodation of the Strengthening Families Programme 10-14: UK Phase I study. Health Educ Res.

[11] Sanchez ZM, Valente JY, Sanudo A, Pereira APD, Cruz JI, Schneider D (2017). The #Tamojunto drug prevention program in Brazilian schools: a randomized controlled trial. Prev Sci.

[12] Carlini ELA, Noto AR, Sanchez ZM, Carlini CMA, Locatelli DP, Abeid LR (2010). VI levantamento nacional sobre o consumo de drogas psicotrópicas entre estudantes do ensino fundamental e médio das redes pública e privada de ensino nas 27 capitais brasileiras - 2010 [Internet].

[13] Saunders JB, Aasland OG, Babor TF, De la Fuente JR, Grant M (1993). Development of the Alcohol Use Disorders Identification Test (AUDIT): WHO collaborative project on early detection of persons with harmful alcohol consumption--II. Addiction.

[14] Lima CT, Freire AC, Silva AP, Teixeira RM, Farrell M, Prince M (2005). Concurrent and construct validity of the AUDIT in an urban Brazilian sample. Alcohol Alcohol.

[15] Babor TF, Robaina K (2016). The Alcohol Use Disorders Identification Test (AUDIT): a review of graded severity algorithms and national adaptations. Int J Alcohol Drug Res.

[16] Associação Brasileira de Empresas de Pesquisas (2020). Critério de Classificação Econômica Brasil: alterações na aplicação do Critério Brasil, válidas a partir de 01/09/2020 [Internet].

[17] Törrönen J, Roumeliotis F, Samuelsson E, Kraus L, Room R (2019). Why are people drinking less than earlier? Identifying and specifying social mechanisms with a pragmatist approach. Int J Drug Policy.

[18] Sonthon P, Janma N, Saengow U (2021). Association between age at first alcohol use and heavy episodic drinking: an analysis of Thailand’s smoking and alcohol drinking behavior survey 2017. PLoS One.

[19] Plens JA, Valente JY, Mari JJ, Ferrari G, Sanchez ZM, Rezende LFM (2022). Patterns of alcohol consumption in Brazilian adults. Sci Rep.

[20] Prates EJS, Prates MLS, Silva LFI, Ferreira GMF, Araújo LMS, Andrade RD (2019). Educational offices with adolescents in the situation of social vulnerability: health promotion, citizenship and empowerment. Expressa Extensão.

[21] Paiva HN, Paiva VS, Paiva PCP (2023). Binge drinking in young adolescents was associated with the consumption of alcoholic beverages by family members and best friends?. ID on line Rev Psicol.

[22] Valente JY, Galvão PPO, Santos MHS, Gubert FA, Sanchez ZM (2024). The role of monitoring skills in mediating the association between parents’ hazardous alcohol consumption and adolescents’ drinking. Soc Psychiatry Psychiatr Epidemiol.

[23] Herrero-Montes M, Alonso-Blanco C, Paz-Zulueta M, Pellico-López A, Ruiz-Azcona L, Sarabia-Cobo C (2022). Excessive alcohol consumption and binge drinking in college students. PeerJ.

[24] Sousa MGM, Castro LC, Malta DC, Gonçalves AMS, Silva FJG, Lima LHO (2024). Factors associated with concurrent alcohol, tobacco and illicit drug use: 2019 National School-Based Health Survey. Cienc Saude Colet.

[25] Tael-Öeren M, Naughton F, Sutton S (2019). The relationship between parental attitudes and children’s alcohol use: a systematic review and meta-analysis. Addiction.

[26] Mitchell S, Campbell R, MacArthur GJ (2022). Parent/caregiver attitudes, motivations and behaviours in relation to alcohol use among offspring aged 13-18 years: a qualitative study. BMC Public Health.

[27] Sanchez ZM, Caetano SC, Galvão PPO, Gubert FA, Rebouças LN, Soares-Santos LE (2025). Does the Brazilian version of the strengthening families program (Famílias Fortes) reduce adolescent substance use and change parental behavior? Evidence from a 2-year follow-up study. BMC Public Health.

[28] Pereda B, Caluda-Perdue N, Levy S, Zhang L, Colder CR (2025). Age of onset of adolescent alcohol use with parental permission and its impact on drinking and alcohol-harms in young adulthood: a longitudinal study. Addict Behav.

[29] Malta DC, Prates EJS, Ferreira ACM, Freitas PC, Oliveira PPV, Gomes CS (2022). Consumption of and exposure to alcoholic beverages among brazilian adolescents: diverse evidence from the 2015 and 2019 national school health surveys. REME Rev Min Enferm.

[30] Bohm MK, Esser MB (2023). Associations between parental drinking and alcohol use among their adolescent children: findings from a national survey of United States parent-child dyads. J Adolesc Health.

[31] Schneider DR, Thurow CF, Franzoloso T, Silva LG, Silva GG, Santos EL (2025). Methodological characteristics of evaluative studies of drug prevention programs in Brazil: scoping review. Rev. Latino-Am. Enfermagem.

[32] Ministério da Saúde (BR) (2014). Programa famílias fortes: versão brasileira adaptada do “Strengthening Families Programme” - SFP 10-14 UK [Internet].

[33] Cimon-Paquet C, Véronneau MH, Mathys C (2024). Beyond the laws: parental monitoring, perceived acceptability of underage drinking and alcohol use among Belgian youth. J Youth Adolesc.

[34] Bowden JA, Bartram A, Harrison NJ, Noris CA, Kim S, Pettigrew S (2024). Australian parents’ attitudes, perceptions and supply of alcohol to adolescents: a national cross-sectional survey. Health Promot Int.

