# Supplementary material for: Parental beliefs, initiation of alcohol consumption, and binge drinking among Brazilian adolescents
Source: Rev Lat Am Enfermagem. 2026 Oct 16;34:e4885. doi: 10.1590/1518-8345.8036.4885 (PMC13674970; doi:10.1590/1518-8345.8036.4885)
Supplement: Translation

# Translation: **Crenças parentais, iniciação do consumo de álcool e *binge drinking* entre adolescentes brasileiros** *

**DOI:** 10.1590/1518-8345.8036.4886

**Authors:** Gyzelda Barros de Sousa, Patrícia Paiva de Oliveira Galvão, Juliana Yurgel Valente, André Ribeiro de Castro, Lidiane Nogueira Rebouças, Zila Van Der Meer Sanchez Dutenhefne, Samir Gabriel Vasconcelos Azevedo, Fabiane do Amaral Gubert

**Affiliations:** 1 Universidade Federal do Ceará, Departamento de Enfermagem, Fortaleza, CE, Brasil.; 2 Bolsista da Coordenação de Aperfeiçoamento de Pessoal de Nível Superior (CAPES), Brasil.; 3 Universidade Federal de São Paulo, Departamento de Medicina Preventiva, Escola Paulista de Medicina, São Paulo, SP, Brasil.; 4 Bolsista do Conselho Nacional de Desenvolvimento Científico e Tecnológico (CNPq), Brasil.; 5 Governo do Estado do Ceará, Secretaria Executiva Sobre Drogas do Ceará, Fortaleza, CE, Brasil.

**Keywords:** Promoção da Saúde, Prevenção Primária, Promoção da Saúde Escolar, Programas Governamentais, Adolescente, Saúde do Adolescente

## Abstract

**Objetivo:**

analisar a associação entre o consumo de álcool por adolescentes e as crenças parentais.

**Método:**

estudo transversal, aninhado em ensaio controlado randomizado por clusters, que avaliou a efetividade de um programa de fortalecimento de vínculos familiares na prevenção ao uso de drogas entre adolescentes. Participaram do estudo 859 famílias com adolescentes entre 10 e 14 anos. Os participantes responderam a um questionário virtual e procederam-se a análises descritivas e regressão multivariada para verificar as associações de interesse.

**Resultados:**

os pais que acreditavam ser mais seguro ensinar os filhos a beberem em casa dobraram as chances de os filhos iniciarem o uso de álcool (OR= 2,10). Já a crença “se um adolescente beber apenas em festas, isso não afetará seu desenvolvimento” quase triplicou (OR= 2,61) as chances de os filhos praticarem *binge drinking*. No estudo, constatou-se que os pais/responsáveis que faziam uso nocivo ou possuíam provável dependência aumentaram em oito vezes as chances de consumo de álcool pelos filhos.

**Conclusão:**

os resultados sugerem que as crenças parentais mais permissivas sobre o consumo de álcool nocivo pelos pais podem elevar significativamente as chances da iniciação precoce e da prática de *binge drinking* na adolescência.

## Destaques

**(1)** Predomina o sexo masculino para o uso de álcool (56,3%) e *binge drinking* (63,3%).

**(2)** 12,3% já iniciaram consumo de álcool, 3,6% já realizam a prática de *binge drinking.*

**(3)** Beber em casa aumenta em 1,97 vezes as chances de iniciarem o uso de álcool.

**(4)** A não aprovação ao uso o que aumenta em 2,37 o consumo de álcool.

**(5)** Os pais que fazem uso nocivo aumentam 8,32 vezes as chances de consumo de álcool.

## Introdução

No Brasil, cerca de 1,5 milhão de adolescentes de 13 ou 14 anos já experimentaram bebida alcoólica e, destes, 13% já haviam consumido alguma droga ilícita em algum momento da vida, com maior prevalência entre estudantes da rede pública^1^. O uso precoce de álcool na adolescência pode estar relacionado às adaptações desta fase, que envolvem a incorporação de novos hábitos e comportamentos, ligados à busca por autonomia e à tomada de decisões^2^. Nesse contexto de mudanças, os adolescentes estão mais expostos a riscos comportamentais, como sexuais de risco, violência, baixo autocuidado ou imprudência no trânsito, quando iniciam o uso dessas substâncias^3^.

A exposição dos adolescentes a esses riscos pode ser influenciada por diversos fatores, como a inexistência de estruturação familiar, exemplos parentais de consumo de substâncias e facilidade de acesso ao álcool e outras drogas^4^.

O consumo de álcool pelos pais exerce papel nuclear na formação dos hábitos e das atitudes dos adolescentes em relação ao consumo de bebidas alcoólicas. Assim, as crenças e atitudes negativas e permissividade na presença dos filhos são fatores que contribuem negativamente para o enfrentamento do álcool na adolescência. A escassez de suporte parental, o consumo de álcool pelos próprios pais, a dificuldade de impor limites são fatores que podem predispor os adolescentes à iniciação ou manutenção do uso de álcool^5^.

A relação entre as crenças dos pais é diretamente associada às práticas parentais. Essas crenças podem ser transmitidas aos filhos por meio de mecanismos de comunicação, como narrativas e instruções, e influenciam a forma como crianças entendem as identidades e o mundo ao redor delas^6^. As crenças influenciam as competências e o desenvolvimento socioemocionais dos adolescentes. Desta forma, essas crenças moldam a forma como os filhos lidam com as emoções e o estresse, habilidade fundamental contra o álcool^7^.

Já a prática parental envolve esforços direcionados ao bem-estar, aprendizado e cultivo de valores dos adolescentes. Essas práticas parentais no desenvolvimento dos adolescentes apontam que os estilos educativos mais compreensivos, de não julgamentos, apoio e aconselhamento são fundamentais para os adolescentes^8^. Presumivelmente, espera-se que práticas parentais positivas contribuam como fatores de proteção para adolescentes em risco para o consumo de álcool. 

Para o enfermeiro conhecer os fatores e as crenças que motivam adolescentes a consumirem álcool e praticarem *binge drinking* ou beber em excesso em determinadas ocasiões, é relevante, pois este profissional atua por meio da educação e promoção da saúde junto às famílias com filhos adolescentes e no ambiente escolar, promovendo o fortalecimento de ações governamentais, como programas de prevenção^9^. Desmistificar crenças não adequadas e investir em estratégias atuantes contra o uso do álcool e *binge drinking* é eixo fundamental para melhorar a saúde do adolescente no Brasil, ao considerar que se trata de um país com várias culturas e formas de perceber o adolescente. 

Diante do exposto, evidências que analisem as crenças parentais e o consumo de álcool na adolescência são relevantes, sobretudo no Brasil, em que a cultura tende a ser permissiva em relação ao consumo de álcool. Nesse contexto, o âmbito familiar constitui ambiente propício para entender o contexto do uso de álcool na adolescência. Assim, objetivou-se analisar a associação entre o consumo de álcool por adolescentes de 10 a 14 anos^1^ e as crenças parentais.

## Método

### Delineamento do estudo

Trata-se de estudo transversal, inserido em um Ensaio Clínico Randomizado (ECR) por *cluster*, projetado para avaliar o efeito do programa governamental de fortalecimento de vínculos familiares, intitulado Famílias Fortes, na prevenção ao consumo de álcool e outras drogas. O estudo analisou os dados da linha de base deste ECR, coletados antes da aplicação da intervenção. A escolha das cidades e a articulação ocorreram durante a implementação do programa, com supervisão e monitoramento do extinto Ministério da Mulher, da Família e dos Direitos Humanos (MMFDH), no ano de 2021. O estudo foi aprovado pelo Comitê de Ética em Pesquisa da Universidade Federal de São Paulo, conforme parecer 4.890.815.

O estudo multicêntrico foi realizado em 12 municípios brasileiros dos estados de São Paulo, Rio de Janeiro, Mato Grosso, Paraíba e Ceará. Os Centros de Assistência Social (CRAS) destes municípios convidaram até 15 famílias compostas por pelo menos um adolescente entre 10 e 14 anos, além de um adulto responsável que pudesse participar dos encontros do programa. As famílias selecionadas deveriam residir até 1 km de distância do CRAS. Não foram incluídas as famílias em que o adolescente estava sob cuidado de terceiros (sem qualquer vínculo familiar), as famílias com necessidades ou desafios muito elevados (como dependência de drogas, ruptura familiar e problemas de comportamento sérios que pudessem interferir no grupo) e aquelas envolvidas em algum programa com o mesmo objetivo ou que já tivessem participado do Programa Famílias Fortes. 

Os CRAS foram randomizados a partir de lista única que continha todos os CRAS dos municípios participantes do estudo (em fase de pactuação e assinatura de convênio com o governo federal). Desta forma, o sorteio não ocorreu por município, mas a partir de uma lista única de CRAS de todos os municípios, utilizando-se do pressuposto da moeda enviesada de Efron, para manter a proporção de 1:1, ou seja, o mesmo número de CRAS controle e CRAS intervenção. O sorteio foi feito a partir do site www.random.org, em duas etapas: 1) sorteio dos CRAS e 2) sorteio das famílias dentro de cada CRAS. A lista das famílias elegíveis foi ofertada pelos CRAS. Ambos os sorteios foram realizados por pesquisador externo ao núcleo principal de pesquisadores. A lista de famílias elegíveis previu uma segunda lista, que foi considerada a lista de espera; assim, quando uma família sorteada não aceitou participar, a primeira família da lista de espera foi convidada.

O grupo controle foi recrutado no formato de “lista de espera pelo programa”, ou seja, os CRAS controle ofertaram a intervenção aos participantes apenas após a finalização da pesquisa, ou seja, um ano depois do início do estudo. Desta forma, os participantes do grupo controle souberam que no futuro receberiam uma intervenção, mas que, no momento, faziam parte do grupo controle e que somente receberiam o programa mediante o encerramento da coleta de dados. Deste modo, ao final, a amostra foi composta por 859 famílias.

Os dados foram coletados entre novembro e dezembro de 2021. Os participantes responderam a um questionário de autopreenchimento virtual, disponibilizado por *smartphones* fornecidos pelos pesquisadores, no próprio CRAS, em local privativo e agendado com antecedência. Algumas coletas foram realizadas nas residências das famílias.

### Instrumentos e medidas

O questionário foi elaborado com base em instrumentos internacionais desenvolvidos para avaliar o *Strengthening Families Programme* (SFP 10-14)^10^, nome original do programa, além de instrumentos validados e previamente utilizados em estudos de avaliação de programas de prevenção ao uso de drogas no Brasil^11^.

A adaptação cultural do programa foi realizada durante os anos de 2014 e 2015 pela Universidade de Brasília. Realizou-se a tradução de todos os materiais do programa, sendo observada a adequação de algumas palavras e práticas culturais para o contexto brasileiro, como a troca de “rugby” e “basquete” por “futebol” e “reunião de família” por “momento em família”. Foram necessárias adaptações visuais, como a atividade “brasão da família”, que foi transformada em “bandeira da família”, implicando a produção de novas figuras para produção da bandeira da família.

Elementos de identidade visual, como imagens e ícones, foram adaptados por artistas brasileiros com características que remetem ao infantil, adaptando cores e formas, deixando a identidade dos manuais mais próxima de outros programas implementados pelo Governo Federal, como o #Tamojunto e o Programa Elos^12^. Procedeu-se, ainda, à revisão dos materiais, propondo alteração em algumas atividades escritas que passaram a ser feitas no formato oral, visto a demanda de participantes com baixo letramento, tendo em vista a sobrecarga dos facilitadores no preparo dos materiais e a demanda dos participantes em levar o material para casa. Assim, criaram-se o material permanente (cartões e cartazes) e os cadernos de atividades para responsáveis e jovens em formato de kit. Mesmo diante das adaptações, o conteúdo programático e o número de encontros não tiveram alterações nos elementos centrais do programa.

A variável desfecho “uso de álcool pelos adolescentes” foi analisada por meio de perguntas extraídas do questionário da Organização Mundial da Saúde (OMS), utilizado pelo Centro Brasileiro de Informações sobre Drogas Psicotrópicas (CEBRID), no VI Levantamento do uso de drogas entre estudantes^12^. Para este estudo, utilizaram-se das seguintes questões para analisar o uso de álcool (sim ou não) e a prática de *binge drinking* na vida (sim ou não): “Você já experimentou alguma bebida alcoólica?” e “Você já tomou 5 doses ou mais de álcool numa mesma ocasião?”.

Já em relação ao “uso de álcool e outras drogas pelos adultos”, aplicou-se o instrumento *Alcohol Use Disorders Identification Test* (AUDIT), que rastreia pessoas em risco de desenvolver transtornos relacionados ao álcool^13^. A versão completa brasileira do AUDIT^14^, que inclui 10 itens, abrange três áreas principais: consumo de álcool (itens 1 a 3), dependência de álcool (itens 4 a 6) e consequências negativas (itens 7 a 10). A pontuação total, que varia de zero a 40 pontos, é frequentemente calculada e utilizada para avaliar o risco associado ao uso de álcool. Essa pontuação permite classificar os participantes em diferentes categorias, como consumo de álcool de baixo risco ou abstinente (0 a 7 pontos), risco de uso perigoso (8 a 15 pontos), uso prejudicial de álcool (16 a 19 pontos) ou dependência de álcool (20 a 40 pontos)^15^.

A variável sobre crenças parentais foi medida por meio de cinco perguntas com respostas dicotômicas (sim ou não): a) “Acho mais seguro ensinar meu filho a beber em casa enquanto ele é adolescente”, b) “Se meu filho não beber, ele será excluído do grupo de amigos”, c) “Ficar bêbado na adolescência é apenas uma fase e passará logo”, d) “Se um adolescente beber apenas em festas, isso não afetará seu desenvolvimento”, e) “Álcool não é droga, é alimento”. 

Os dados sociodemográficos incluíram sexo, escolaridade, idade e status socioeconômico, este último coletado por meio da escala da Associação Brasileira de Empresas de Pesquisa (ABEP)^16^. Pontuações mais altas indicam melhor posição econômica, e as classes socioeconômicas são classificadas de A (mais alta) a E (mais baixa), e o escore é gerado a partir de informações sobre bens de consumo e escolaridade do chefe da família. De acordo com a ABEP, as classes D/E podem ser agrupadas. Todas as variáveis utilizadas neste estudo foram coletadas no momento inicial (linha de base) e, portanto, não foram randomizadas nem influenciadas pela intervenção.

Os grupos controle e intervenção foram submetidos ao mesmo questionário virtual e áudio-guiado em três momentos: antes do início da intervenção, seis meses depois e 12 meses depois. O questionário foi disponibilizado aos participantes por meio de *smartphones*, por pesquisadores vinculados às universidades promotoras do estudo, sem a presença do facilitador do programa. O preenchimento foi anônimo e o mesmo questionário foi aplicado nos três momentos de coleta de dados; no entanto, aos questionários do *follow-up* serão acrescentadas algumas perguntas referentes à experiência de participar do programa. O questionário foi previamente preenchido pelos pesquisadores de campo com um código de município e CRAS. Este código permitiu análises de *cluster* (multinível) em dois níveis (município e serviço). Destacam-se experiências prévias da equipe com este modelo de código sigiloso para pareamento no tempo. Ao código individual foi somado um código de família e de CRAS, para permitir pareamento entre adolescentes e identificação do cluster de CRAS.

Os dados foram coletados por questionários estruturados, virtuais, anônimos e de autopreenchimento planejado, a partir de referenciais internacionais utilizados para avaliação SFP 10-14^12^. A aplicação do instrumento ocorreu em local privativo dentro do CRAS ou em estrutura indicada pelo serviço. Os pesquisadores disponibilizaram tablets ou computadores para o preenchimento por parte das famílias. Ao preencher, pais e adolescentes ficaram em local separado, a fim de preservar a confidencialidade, o sigilo e, principalmente, pelos participantes adolescentes, evitando o sentimento de que os dados seriam socializados com os pais.

Pesquisas com muitos sujeitos e seguimento de tempo tendem a perdas do seguimento. Considerando a dimensão do ensaio controlado apresentado, adotou-se a imputação múltipla dos dados, a fim de diminuir viés e aumentar a fidedignidade dos resultados. Ao longo da pesquisa, adotaram-se técnicas motivacionais e comportamentais com as famílias, sobretudo com os adolescentes, estimulando o engajamento no programa e preenchimento dos questionários.

Ao considerar a sensibilidade das temáticas abordadas, algumas iniciativas foram adotadas, a fim de garantir redução no viés de informação, os procedimentos de campo foram: 1) anunciar aos sujeitos que receberam um questionário sobre saúde e comportamentos; 2) um ou dois pesquisadores treinados estiveram presentes durante o processo de preenchimento dos questionários e a presença de qualquer profissional do CRAS não foi permitida neste momento de coleta; 3) os questionários foram preenchidos de forma eletrônica em smartphones individuais para evitar contato entre os pesquisadores e os questionários preenchidos, garantindo, assim, maior sigilo da informação; 4) os pesquisadores de campo evidenciaram mais de uma vez que o questionário é anônimo e os dados não foram ou serão acessados por ninguém além dos pesquisadores; 5) jovens responderam aos questionários em salas separadas, acompanhados de um membro da equipe de pesquisa.

### Análise estatística

As variáveis estudadas foram inicialmente descritas de acordo com a natureza das medidas, sendo sumarizadas por meio de números absolutos e prevalências. No segundo momento, realizou-se teste de hipótese para avaliar a associação entre as variáveis de interesse e o uso de álcool pelos adolescentes mediante o uso de teste qui-quadrado. Posteriormente, considerou-se o modelo inicial de regressão logística multivariada, incluindo as variáveis explicativas que apresentaram valor de p ≤ 0,20 na regressão univariada. Em seguida, empregou-se procedimento retroativo para remover as variáveis explicativas com p > 0,05, com objetivo de obter um modelo final para cada variável de resposta, as respectivas razões de chance (OR), o intervalo de confiança (IC) de 95% e os valores de p. O nível de significância adotado foi de 5%.

As análises foram realizadas nas duas amostras: “*intention to treat*” (ITT), incluídos todos os sujeitos que tenham sido randomizados para participar do estudo, de acordo com o grupo original de alocação, independentemente de terem dados perdidos; bem como “Casos Completos” (CC), em que foram consideradas apenas as observações com dados completos no tempo inicial e nos acompanhamentos; portanto, nessa amostra, casos com valores ausentes foram excluídos.

As medidas de associação foram analisadas via *Generalized Linear Mixed Models* (GLMM), que calcula o nível de dependência entre os dados. Ao considerar a estrutura hierárquica dos dados, utilizaram-se modelos multiníveis para verificar simultaneamente diferenças nas prevalências de consumo entre os tempos e entre os grupos, ponderando escalas como nível de modelagem^12^. Para construção, organização e apresentação metodológica, o estudo está alinhado ao *checklist Strengthening the Reporting of Observational Studies in Epidemiology* (STROBE).

## Resultados

A Tabela 1 aponta que a média de idade dos adolescentes foi de 12,6 anos e, para os pais, 39,5 anos, sendo a maioria pertencente ao status socioeconômico na categoria D/E (73,2%). Quanto ao uso de drogas na vida, 12,3% dos adolescentes relataram que consumiram álcool e 3,6% realizaram a prática de *binge drinking*. Já os pais, a maioria (96,3%) apresentou consumo de baixo risco de álcool. No tocante às crenças parentais sobre o uso de álcool pelos adolescentes, destaca-se que 10,5% acreditavam ser mais seguro ensinar o filho a beber em casa e 20,8% afirmaram que os adolescentes que bebem somente em festas não terão o desenvolvimento afetado.

**Tabela 1:** Características sociodemográficas dos adolescentes e adultos participantes do estudo (n = 859). Fortaleza, CE, Brasil, 2021

Variáveis	Adolescentes		Adultos	
	(n= 884)		(n= 884)	
	n*	%^†^	n*	%^†^
**Sexo**				
Masculino	414	48,2	72	8,4
Feminino	439	51,1	782	91,0
Outros	6	0,7	5	0,6
**Idade (média DP)** ^‡^	12,6±1,24		39,50±8,15	
**Status socioeconômico**				
A	---	---	8	0,9
B	---	---	33	3,8
C	---	---	190	22,1
D/E	---	---	628	73,2
**Uso de drogas na vida (adolescentes)**				
Álcool	104	12,3	---	---
Prática de *binge drinking*	30	3,6	---	---
**Consumo de álcool pelos responsáveis**				
Baixo risco ou abstêmios	---	---	827	96,3
Risco	---	---	21	2,4
Uso nocivo ou provável dependência	---	---	11	1,3
Crenças sobre o uso de drogas				
Seguro Beber em Casa^§^	---	---	89	10,5
Ser Excluído^||^	---	---	149	17,5
Bêbado na Adolescência^¶^	---	---	116	13,7
Beber em Festas**	---	---	177	20,8
Álcool não é Droga^††^	---	---	45	5,3

*n = Tamanho da amostra; ^†^Valor percentual; ^‡^Valor médio da idade; ^§^Acho mais seguro ensinar meu filho a beber em casa enquanto ele é adolescente; ^||^Se meu filho não beber, ele será excluído do grupo de amigos; ^¶^Ficar bêbado na adolescência é apenas uma fase e passará logo; **Se um adolescente beber apenas em festas, isso não afetará seu desenvolvimento; ^††^Álcool não é droga, é alimento

A Tabela 2 mostra que o uso de álcool, assim como a prática de *binge drinking,* é mais prevalente em adolescentes do sexo masculino. Da mesma forma, a idade é um parâmetro relevante e evidencia-se que adolescentes de 13 e 14 anos fazem mais consumo quando comparados aos de 11 e 12 - isso foi comprovado estatisticamente. Na análise das crenças parentais, para os adolescentes que consumiam bebidas alcoólicas, 19,3% tinham pais que acreditavam ser mais seguro ensinar o filho a beber em casa enquanto ele é adolescente (p=0,038). Já as crenças “ficar bêbado na adolescência é apenas uma fase e passará logo” e “se um adolescente beber apenas em festas, isso não afetará seu desenvolvimento” obtiveram associação com a prática de *binge drinking* pelos adolescentes (p=0,037 e p=0,010, respectivamente). 

**Tabela 2:** Distribuição do uso de álcool e da prática de *binge drinking* pelos adolescentes, segundo as variáveis sociodemográficas, as crenças parentais e o consumo de álcool dos pais. Fortaleza, CE, Brasil, 2021

	Uso de álcool na vida					***Binge drinking* na vida**				
Variáveis	Não		Sim		P^‡^	Não		Sim		P^‡^
	n*	%^†^	n*	%^†^		n*	%^†^	n^*^	%^†^	
**Sexo do adolescente**										
Masculino	352	47,3	58	55,8	0,238	382	47,3	19	63,3	0,212
Feminino	388	52,1	45	43,3		420	52,0	11	36,7	
Outros	5	0,6	1	0,9		6	0,7	0	-	
**Idade do adolescente**										
10-12 anos	365	49	20	19,2	**<0,001**	370	45,8	9	30	0,088
13-14 anos	380	51	84	80,8		438	54,2	21	70	
**Associação Brasileira de Empresas de Pesquisas**										
A	7	0,9	0	-	0,684	8	1	0	-	0,813
B	28	3,8	5	4,8		31	3,8	1	3,3	
C	163	21,9	25	24		181	22,4	5	16,7	
D/E	547	73,4	74	71,2		588	72,8	24	80	
**Crenças dos pais**										
Seguro Beber em Casa^§^	71	80,7	17	19,3	**0,038**	84	97,7	2	2,3	0,603
Ser Excluído^||^	129	87,2	19	12,8	0,771	141	95,9	6	4,1	0,669
Bêbado na Adolescência^¶^	97	83,6	19	16,4	0,159	106	93	8	7,0	**0,037**
Beber em Festas**	152	86,9	23	13,1	0,716	163	93,1	12	6,9	**0,010**
Álcool não é Droga^††^	37	86,1	6	13,9	0,741	42	95,5	2	4,5	0,731
**Consumo de álcool pelos pais**										
Consumo de baixo risco ou abstêmios	720	88,1	97	11,9	**<0,001**	778	96,4	29	3,6	0,769
Consumo de risco	20	95,2	1	4,8		19	95	1	5,0	
Uso nocivo ou provável dependência	5	45,5	6	54,5		11	100	0	-	

*n = Tamanho da amostra; ^†^Valor percentual; ^‡^p = p valor no nível de 5% (p ≤ 0,05); ^§^Acho mais seguro ensinar meu filho a beber em casa enquanto ele é adolescente; ^||^Se meu filho não beber, ele será excluído do grupo de amigos; ^¶^Ficar bêbado na adolescência é apenas uma fase e passará logo; **Se um adolescente beber apenas em festas, isso não afetará seu desenvolvimento; ^††^Álcool não é droga, é alimento

A Tabela 3 destaca a associação entre o consumo de álcool pelos pais e as crenças em relação ao consumo de álcool e prática de *binge drinking* pelos filhos. Observou-se que os pais que “acham mais seguro ensinar seus filhos a beberem em casa” dobraram as chances de os filhos iniciarem o uso de álcool (OR= 2,10). Em relação aos pais que relataram acreditar que “se um adolescente beber apenas em festas, isso não afetará seu desenvolvimento”, quase triplicaram (OR= 2,61) as chances de os filhos praticarem *binge drinking*. Outro dado importante foi que os pais/responsáveis que faziam uso nocivo ou possuíam provável dependência de álcool aumentaram em oito vezes as chances de consumo de álcool pelos filhos (OR= 8,91).

**Tabela 3:** Associação entre iniciação ao uso do álcool, *binge drinking* (adolescentes) e crenças parentais e consumo de álcool pelos pais, de acordo com estimação de OR* e intervalo de confiança 95%^†^ (N = 854). Fortaleza, CE, Brasil, 2021

	Regressão Logística Univariada						Regressão Logística Multivariada					
	Uso de álcool na vida			**Prática de *binge drinking***			Uso de álcool na vida			**Prática de *binge drinking***		
Variáveis	OR*	95%IC^†^	p^‡^	OR*	95%IC^†^	p^‡^	OR*	95%IC^†^	p^‡^	OR*	95%IC^†^	p^‡^
**Crenças dos pais**												
Seguro Beber em Casa^§^	1,82	[1,03;3,24]	0,041	0,68	[0,16;2,93]	0,605	2,10	[1,04;3,53]	0,036	---	---	--
Ser Excluído^||^	1,08	[0,63;1,85]	0,771	1,22	[0,49;3,05]	0,669	---	---	--	---	---	--
Bêbado na Adolescência^¶^	1,47	[0,86;2,53]	0,161	2,37	[1,03;5,47]	0,042	---	---	---	---	---	--
Beber em Festas**	1,10	[0,67;1,80]	0,716	2,61	[1,23;5,53]	0,012	---	---	---	2,61	[1,23;5,53]	0,012
Álcool não é Droga^††^	1,16	[0,48;2,82]	0,741	1,29	[0,30;5,61]	0,732	---	---	--	---	---	--
**Consumo de álcool pelos pais**												
Consumo de baixo risco ou abstêmios	ref			ref			ref			ref		
Consumo de risco	0,37	[0,05; 2,80]	0,336	1,41	[0,18; 10,91]	0.741	---	---	---	---	---	---
Uso nocivo ou provável dependência	8,91	[0,67; 29,74]	<0.001	---	---	---	6,41	[1,84;22,34]	0,004	---	---	---

^*^OR *= Odds Ratio*; ^†^Intervalo de Confiança de 95%; ^‡^p = p valor no nível de 5% (p ≤ 0,05); ^§^Acho mais seguro ensinar meu filho a beber em casa enquanto ele é adolescente; ^||^Se meu filho não beber, ele será excluído do grupo de amigos; ^¶^Ficar bêbado na adolescência é apenas uma fase e passará logo; **Se um adolescente beber apenas em festas, isso não afetará seu desenvolvimento; ^††^Álcool não é droga, é alimento

## Discussão

O estudo identificou que algumas crenças, como ensinar o filho a beber em casa e achar que beber em festas não é problema, estão associadas ao início do consumo do álcool de forma precoce e à prática de *binge drinking* na adolescência. Além disso, identificou-se que os pais que apresentaram uso nocivo de álcool ou possuíam provável dependência aumentaram em até oito vezes as chances de os adolescentes iniciarem o uso de bebidas alcoólicas, comparado com aqueles que não faziam uso nocivo. A relevância desses dados reitera a necessidade de o enfermeiro trabalhar com pais e adolescentes sobre crenças.

Os adolescentes neste estudo tiveram média de idade de 12,6 anos e consumo em cerca de 12,3% dos participantes. Essa realidade é muito similar ao contexto nacional, uma vez que a média é por volta de 13 anos^17^. Quando se compara com países de perfis socioeconômicos semelhantes, como o México e a Tailândia, é ainda mais comum a experimentação do álcool pelos adolescentes em idade precoce, que pode alcançar a prevalência de 18% a 60% para essa mesma faixa etária^18^.

Em relação aos adultos que consomem álcool, neste estudo, 5,8% faziam uso nocivo ou possuíam provável dependência ao consumo de álcool, mas é válido destacar que, nesta amostra, 91% dos adultos eram mulheres. Socialmente, as mulheres estão em maior vulnerabilidade e, no consumo de álcool, isso revela cenário preocupante com consequências, enfaticamente, na saúde mental. Apesar disso, o cenário brasileiro é caracterizado de forma oposta, pois percebe-se que, dos bebedores pesados, a prevalência foi de 3,2% entre os homens e de 0,6% entre mulheres^19^. 

O presente estudo encontrou forte associação entre o consumo de álcool pelos pais e consumo de álcool pelos adolescentes. Identificou-se que os pais/responsáveis que faziam uso nocivo ou possuíam provável dependência de álcool aumentaram em mais de oito vezes as chances de consumo de álcool pelos adolescentes. De maneira análoga, estudo na Região Sul do Brasil, com 1.170 adolescentes, apresentou que 48% dos que faziam uso relataram que os pais faziam uso de bebida alcoólica e que 10,5% destes pais apresentaram algum problema com álcool nos últimos 30 dias^20^. Outro estudo no país apresentou que o uso de bebidas alcoólicas em casos familiares de consumo por mãe e pai, esteve associado com o beber em *binge drinking* pelos adolescentes^21^. Essa correlação do consumo dos jovens pode ser explicada pela relação parental e exemplo evidenciado na família; assim, os jovens espelham o comportamento no modelo de criação a que são submetidos^22^. 

Em relação às crenças parentais sobre o consumo de álcool pelos filhos, verificou-se que os adultos que responderam considerar mais seguro ensinar o filho a beber em casa aumentaram consideravelmente as chances de os filhos iniciarem precocemente o consumo da bebida, apresentando razão de 1,97 mais chances de início desse consumo. Além disso, 20,8% dos adultos acreditaram que, se o adolescente beber somente em festas, esta prática não afetará o desenvolvimento dele. Esta crença, no contexto do estudo, pode quase dobrar as chances de os filhos praticarem *binge drinking*. A prática do consumo em festas foi apontada como disparadora para o consumo excessivo, além de o ambiente de festa ser indicado como associação ao local do primeiro uso de álcool ou outras drogas. A prática de *binge drinking* influencia negativamente os processos de aprendizagem do adolescente, predispõe à toxicodependência, ao uso de cigarro e drogas ilícitas e a crises de abstinência^23^.

Em famílias cujos pais fazem uso de substâncias psicoativas, observa-se maior probabilidade de consumo entre os filhos. Presume-se que a associação significativa entre a prática de pais/responsáveis que bebem e o policonsumo entre os escolares possa refletir a influência parental sobre os adolescentes^24^. O ambiente familiar constitui o principal grupo social do adolescente durante o processo de crescimento, podendo atuar como fator desencadeador para o consumo de substâncias psicoativas. A relação entre o comportamento do jovem e o dos pais pode estar associada à reprodução de padrões observados no convívio familiar.

Já os pais/responsáveis que responderam positivamente que “se um adolescente beber apenas em festas, isso não afetará seu desenvolvimento” resultaram no aumento de 2,61 vezes nas chances de iniciação ao uso de álcool pelos adolescentes. Enquanto aqueles que responderam “Ficar bêbado na adolescência é apenas uma fase e passará logo”, incidiu aumento de 2,37 vezes a chance do consumo de álcool. De maneira semelhante aos achados, em revisão sistemática, apontou-se que atitudes parentais menos restritivas em relação ao consumo de álcool entre jovens estiveram associadas a maiores taxas de iniciação no uso de álcool (OR) = 1,45, frequência de consumo (OR = 1,52) e episódios de embriaguez (OR = 1,58)^25^.

O comportamento parental em relação ao uso de álcool está ligado a fatores como ambiente familiar, estilo de parentalidade, limites e influências culturais. Pais demonstram consciência dos riscos do álcool e desaprovam o uso por jovens, mas reconhecem a presença inevitável na cultura. Assim, muitos adotam postura equilibrada e relutantemente receptiva, buscando conciliar valores pessoais com a promoção da autonomia dos filhos e a adaptação às normas sociais^26^.

O programa apresentou um aumento significativo na capacidade dos pais de estabelecer regras claras sobre o uso de drogas pelos filhos após 24 meses de acompanhamento. A literatura sobre prevenção ao uso de substâncias mostra de forma consistente que regras parentais bem definidas e firmes constituem um importante fator de proteção contra o início e a progressão do consumo entre adolescentes. Essas regras ajudam a criar limites objetivos, permitindo que os jovens compreendam as expectativas familiares e sociais em relação ao uso de drogas, além de reforçarem normas comportamentais que desencorajam esse consumo. Diante desse cenário, os efeitos do programa na redução da negligência parental e no fortalecimento das práticas de definição de regras podem exercer impacto substancial na prevenção do uso de substâncias. Pesquisas futuras devem investigar, por meio de modelos de mediação, se essas mudanças positivas nos comportamentos parentais, juntamente com a diminuição da negligência, influenciam diretamente a prevenção do uso de drogas entre adolescentes^27^.

Vale destacar que a permissividade dos pais em relação ao uso de álcool por adolescentes está associada a danos a longo prazo, independentemente da idade de início do consumo^28^. Diante disso, o enfermeiro assume papel estratégico na prevenção, atuando na identificação e desconstrução de crenças que normalizam o consumo precoce de álcool. É fundamental que o enfermeiro desenvolva ações educativas planejadas, especialmente no ambiente escolar, com o objetivo de sensibilizar pais, alunos e a comunidade sobre os riscos envolvidos. Essas intervenções contribuem para redução de fatores de risco e promoção de ambientes mais protetores para os adolescentes. 

Com base no exposto, ao identificar o perfil de consumo entre adolescentes, é importante considerar que o uso de álcool pode favorecer comportamentos violentos, brigas, causando desinibição e tornando os adolescentes mais vulneráveis^29^. Portanto, conhecer o contexto das crenças parentais é relevante para o cuidado em saúde, principalmente porque cada estilo parental tem implicações significativas no desenvolvimento dos filhos e pode influenciar o bem-estar emocional, social e cognitivo ao longo da vida.

Intervenções direcionadas aos pais que fortalecem o monitoramento, a comunicação eficaz e o apoio emocional têm se mostrado eficazes. Essas estratégias reduzem diretamente a disponibilidade e a acessibilidade do álcool para adolescentes. Além disso, atuam de forma indireta, influenciando comportamentos e percepções adultas, ao promover ambientes regulatórios mais rigorosos e normas parentais mais saudáveis, ambos fatores associados à redução do consumo de álcool por menores de idade^30^ ^)-(^ ^31^.

É nesse contexto que os programas de prevenção baseados em evidência, como o Programa Famílias Fortes, proposto pelo Governo Federal brasileiro, propõem intervenções que atuem na qualidade das relações familiares, desenvolvendo apego seguro entre crianças e pais, incluindo disciplina afetiva, supervisão e monitoramento dos pais. O desenvolvimento destas habilidades ocorre de maneira programada, planejada e sistemática, favorecendo as famílias para aprender e treinar essas habilidades de forma interativa, sendo a forma mais efetiva de aprendizado^32^.

As evidências indicam que os pais tendem a relatar níveis mais elevados de monitoramento parental do que seus filhos, e que essas discrepâncias são maiores para comportamentos relacionados à definição de regras do que para solicitações parentais. Além disso, ao longo do tempo, tanto pais quanto jovens relatam diminuição das regras parentais, embora apenas os pais relatem uma redução nas solicitações. Diante disso, torna-se essencial examinar as percepções dos jovens sobre o monitoramento parental em relação ao consumo de álcool, especialmente entre adolescentes com diferentes padrões de uso (isto é, abstêmios e consumidores), sobretudo quando eles atingem a idade mínima legal para beber. Além disso, regras específicas sobre o consumo de álcool têm se mostrado eficazes na redução do uso em estudos de intervenção^33^ ^)-(^ ^34^.

Dentre as limitações do estudo, destaca-se o fato de ter sido realizado apenas em doze cidades brasileiras, o que exige cautela ao se generalizarem os resultados para a população brasileira, em razão da ampla diversidade geográfica e cultural. Além disso, os questionários foram aplicados em ambiente da rede de apoio social, o que pode ter influenciado as respostas, devido à presença dos colegas e ao contexto social. Ainda que a coleta de dados tenha ocorrido de forma separada dos pais, em determinadas etapas, é possível que os adolescentes tenham sentido algum nível de julgamento ou recriminação, mesmo que apenas no imaginário deles.

Os resultados oferecem subsídios importantes para os profissionais que atuam na área da saúde do adolescente, ao apontarem as crenças parentais como questão relevante de saúde pública. Torna-se, portanto, fundamental a promoção de intervenções precoces que visem à prevenção de comportamentos de risco. No contexto brasileiro, a cultura permissiva em relação ao consumo de álcool na adolescência, aliada ao frágil controle sobre a venda de bebidas alcoólicas, reforça a necessidade urgente de investimentos em pesquisas que aprofundem a compreensão dessas vulnerabilidades.

## Conclusão

O estudo apontou que as crenças parentais permissivas, como consumir bebidas alcoólicas em casa e em festas e o uso nocivo de álcool pelos pais, podem aumentar consideravelmente as chances da iniciação precoce do uso de álcool pelos filhos e a prática de *binge drinking*. Desta forma, o consumo de bebida alcoólica pelos pais é fator agravante à saúde do adolescente, acarretando prejuízos quanto às práticas de vida.

Assim, é importante o desenvolvimento de programas de prevenção para as famílias com membros ainda na fase da adolescência inicial, e que não somente esclareçam sobre as consequências do uso precoce de bebidas alcoólicas, mas que trabalhem práticas sobre educação parental, modificando crenças parentais consideradas de risco para o uso de álcool na adolescência, implicando na prevenção. Destacam-se, por fim, as várias possibilidades de ações para o enfermeiro trabalhar fatores de proteção, ao incluir crenças do uso do álcool e prática de *binge drinking* com pais e adolescentes, no escopo de prevenção. Ademais, estudos que avaliem as crenças dos pais e os conhecimentos destes a respeito dos agravos que a bebida alcoólica pode produzir na vida dos adolescentes são importantes para nortear o desenvolvimento desses programas.

## Data Availability

Os conjuntos de dados relacionados a este artigo estarão disponíveis mediante solicitação ao autor correspondente.

# Translation: Creencias parentales, inicio del consumo de alcohol y consumo excesivo de alcohol entre adolescentes brasileños*

**DOI:** 10.1590/1518-8345.8036.4884

**Authors:** Gyzelda Barros de Sousa, Patrícia Paiva de Oliveira Galvão, Juliana Yurgel Valente, André Ribeiro de Castro, Lidiane Nogueira Rebouças, Zila Van Der Meer Sanchez Dutenhefne, Samir Gabriel Vasconcelos Azevedo, Fabiane do Amaral Gubert

**Affiliations:** 1 Universidade Federal do Ceará, Departamento de Enfermagem, Fortaleza, CE, Brasil.; 2 Becaria (o) de la Coordenação de Aperfeiçoamento de Pessoal de Nível Superior (CAPES), Brasil.; 3 Universidade Federal de São Paulo, Departamento de Medicina Preventiva, Escola Paulista de Medicina, São Paulo, SP, Brasil.; 4 Becaria (o) del Conselho Nacional de Desenvolvimento Científico e Tecnológico (CNPq), Brasil.; 5 Governo do Estado do Ceará, Secretaria Executiva Sobre Drogas do Ceará, Fortaleza, CE, Brasil.

**Keywords:** Promoción de la Salud, Prevención Primaria, Promoción de la Salud Escolar, Programas de Gobierno, Adolescente, Salud del Adolescente

## Abstract

**Objetivo:**

analizar la asociación entre el consumo de alcohol por adolescentes y las creencias parentales.

**Método:**

estudio transversal, integrado en un ensayo controlado aleatorizado por conglomerados, que evaluó la eficacia de un programa para fortalecer los lazos familiares en la prevención del consumo de drogas entre adolescentes. El estudio incluyó a 859 familias con adolescentes de entre 10 y 14 años. Los participantes respondieron a un cuestionario virtual y realizaron análisis descriptivos y regresión multivariante para verificar asociaciones de interés.

**Resultados:**

los padres que creían que era más seguro enseñar a sus hijos a beber en casa duplicaron las probabilidades de que sus hijos empezaran a consumir alcohol (OR = 2,10). Por otro lado, la creencia de que “si un adolescente solo bebe en las fiestas, no afectará su desarrollo” casi triplicó (OR= 2,61) las probabilidades de que los niños practicaran el consumo excesivo de alcohol. En el estudio, se encontró que los padres o tutores que hacían uso nocivo o tenían probable dependencia aumentaban ocho veces las probabilidades de consumo de alcohol por parte de sus hijos.

**Conclusión:**

los resultados sugieren que creencias parentales más permisivas sobre el consumo de alcohol dañino por parte de los padres pueden aumentar significativamente las probabilidades de inicio temprano y de consumo excesivo de alcohol en la adolescencia.

## Destacados

**(1)** Predomina el sexo masculino en el consumo de alcohol (56,3 %) y el consumo excesivo de alcohol (63,3 %).

**(2)** El 12,3% ya ha empezado a beber alcohol, el 3,6% ya ha bebido en exceso.

**(3)** Beber en casa aumenta las probabilidades de empezar a beber en 1,97 veces.

**(4)** No aprobar el consumo de alcohol, que aumenta el consumo de alcohol en 2,37.

**(5)** Los padres que lo usan de forma dañina aumentan las probabilidades de consumo de alcohol en 8,32 veces.

## Introducción

En Brasil, alrededor de 1,5 millones de adolescentes de 13 o 14 años ya han probado bebidas alcohólicas y, de estos, el 13% ya había consumido alguna droga ilícita en algún momento de sus vidas, con una mayor prevalencia entre los estudiantes del sistema público de enseñanza^1^. El consumo temprano de alcohol en la adolescencia puede estar relacionado con las adaptaciones de esta fase, que implican la incorporación de nuevos hábitos y comportamientos, vinculados a la búsqueda de autonomía y la toma de decisiones^2^. En este contexto de cambios, los adolescentes están más expuestos a riesgos conductuales, como el riesgo sexual, la violencia, el bajo autocuidado o la imprudencia en el tráfico, cuando empiezan a consumir estas sustancias^3^.

La exposición de los adolescentes a estos riesgos puede estar influida por varios factores, como la falta de estructura familiar, ejemplos parentales de consumo de sustancias y facilidad de acceso al alcohol y otras drogas^4^.

El consumo de alcohol por parte de los padres desempeña un papel central en la formación de hábitos y actitudes de los adolescentes en relación con el consumo de bebidas alcohólicas. Por tanto, las creencias y actitudes negativas y la permisividad en presencia de niños son factores que contribuyen negativamente a afrontar el alcohol en la adolescencia. La falta de apoyo parental, el consumo de alcohol por parte de los propios padres y la dificultad para imponer límites son factores que pueden predisponer a los adolescentes a iniciar o mantener el consumo de alcohol^5^.

La relación entre las creencias de los padres está directamente asociada con las prácticas de crianza. Estas creencias pueden transmitirse a los niños a través de mecanismos de comunicación, como narrativas e instrucciones, e influyen en la forma en que los niños entienden las identidades y el mundo que les rodea^6^. Las creencias influyen en las habilidades socioemocionales y el desarrollo de los adolescentes. Así, estas creencias moldean la forma en que los niños afrontan las emociones y el estrés, una habilidad fundamental contra el alcohol^7^.

La práctica parental, en cambio, implica esfuerzos orientados al bienestar, el aprendizaje y la promoción de los valores de los adolescentes. Estas prácticas parentales en el desarrollo de los adolescentes indican que estilos educativos más completos, la ausencia de juicios, el apoyo y la orientación son fundamentales para los adolescentes^8^. Presumiblemente, se espera que las prácticas de crianza positivas contribuyan como factores protectores para los adolescentes en riesgo de consumo de alcohol.

Para las enfermeras, conocer los factores y creencias que motivan a los adolescentes a consumir alcohol y a beber en exceso o en exceso en determinadas ocasiones es relevante, ya que este profesional trabaja a través de la educación y la promoción de la salud con familias con niños adolescentes y en el entorno escolar, promoviendo el fortalecimiento de las acciones gubernamentales. como programas de prevención^9^. Desmitificar creencias inapropiadas e invertir en estrategias para actuar contra el consumo de alcohol y el consumo excesivo de alcohol es un eje fundamental para mejorar la salud adolescente en Brasil, teniendo en cuenta que es un país con diversas culturas y formas de percibir a los adolescentes.

En vista de lo anterior, la evidencia que analiza las creencias parentales y el consumo de alcohol en la adolescencia es relevante, especialmente en Brasil, donde la cultura tiende a ser permisiva en relación con el consumo de alcohol. En este contexto, el entorno familiar es favorable para entender el contexto del consumo de alcohol en la adolescencia. Por tanto, el objetivo de este estudio fue analizar la asociación entre el consumo de alcohol en adolescentes de 10 a 14 años^1^ y las creencias de los padres.

## Método

### Diseño del estudio

Se trata de un estudio transversal, insertado en un Ensayo Clínico Randomizado (ECR) basado en conglomerados diseñado para evaluar el efecto del programa gubernamental para fortalecer los lazos familiares, titulado Familias Fuertes, en la prevención del consumo de alcohol y otras drogas. El estudio analizó los datos de referencia de este ECR, recogidos antes de la aplicación de la intervención. La elección de las ciudades y la articulación se llevaron a cabo durante la implementación del programa, con supervisión y seguimiento por parte del extinto Ministerio de Mujer, Familia y Derechos Humanos (MMFDH), en 2021. El estudio fue aprobado por el Comité de Ética de la Investigación de la Universidade Federal de São Paulo, según el dictamen 4.890.815.

El estudio multicéntrico se llevó a cabo en 12 municipios brasileños de los estados de São Paulo, Río de Janeiro, Mato Grosso, Paraíba y Ceará. Los Centros de Asistencia Social (CRAS) de estos municipios invitaban hasta 15 familias compuestas por al menos un adolescente de entre 10 y 14 años, además de un adulto responsable que podía participar en las reuniones del programa. Las familias seleccionadas deben vivir hasta a 1 km del CRAS. No se incluyeron las familias en las que el adolescente estaba bajo el cuidado de terceros (sin ningún vínculo familiar), las familias con necesidades o desafíos muy elevados (como adicción a las drogas, la ruptura familiar y graves problemas de comportamiento que pudieran interferir con el grupo) ni aquellas involucradas en un programa con el mismo objetivo o que ya hubieran participado en el Programa Familias Fuertes.

Los CRAS se seleccionaron aleatoriamente a partir de una única lista que contenía todos los CRAS de los municipios participantes en el estudio (en la fase de acuerdo y firma de un acuerdo con el gobierno federal). Así, el sorteo no se produjo por municipio, sino a partir de una única lista de CRAS de todos los municipios, usando la suposición de la moneda Efron sesgada, para mantener la proporción de 1:1, es decir, el mismo número de control CRAS e intervención CRAS. El sorteo se realizó desde la página web de www.random.org, en dos fases: 1) sorteo del CRAS y 2) sorteo de las familias dentro de cada CRAS. La lista de familias elegibles fue ofrecida por la CRAS. Ambos sorteos fueron realizados por un investigador externo al grupo principal de investigadores. La lista de familias elegibles incluía una segunda lista, que se consideraba lista de espera; Así, cuando una familia seleccionada no aceptaba participar, se invitaba a la primera familia en la lista de espera.

El grupo de control fue reclutado en el formato de “lista de espera para el programa”, es decir, el CRAS de control ofrecía la intervención a los participantes solo después de finalizar la investigación, es decir, un año después del inicio del estudio. De este modo, los participantes del grupo de control sabían que en el futuro recibirían una intervención, pero que, en ese momento, formaban parte del grupo de control y que solo recibirían el programa tras finalizar la recogida de datos. Así, al final, la muestra estaba compuesta por 859 familias.

Los datos se recopilaron entre noviembre y diciembre de 2021. Los participantes respondieron a un cuestionario virtual de autocompletado, disponible mediante *smartphones* proporcionados por los investigadores, en la propia CRAS, en un lugar privado y programado con antelación. Algunas colectas se realizaban en las casas de las familias.

### Instrumentos y medidas

El cuestionario fue desarrollado basándose en instrumentos internacionales desarrollados para evaluar el Programa de Fortalecimiento de Familias (SFP 10-14)^10^, el nombre original del programa, además de instrumentos validados previamente utilizados en estudios para evaluar programas de prevención del consumo de drogas en Brasil^11^.

La adaptación cultural del programa se llevó a cabo durante los años 2014 y 2015 por la Universidad de Brasilia. Se tradujeron todos los materiales del programa, y se observó la adecuación de algunas palabras y prácticas culturales para el contexto brasileño, como el intercambio de “rugby” y “baloncesto” por “fútbol” y “reunión familiar” por “momento familiar”. Fueron necesarias adaptaciones visuales, como la actividad del “escudo familiar”, que se transformó en la “bandera familiar”, implicando la producción de nuevas figuras para la fabricación de la bandera familiar.

Elementos de identidad visual, como imágenes e iconos, fueron adaptados por artistas brasileños con características que hacen referencia a la infancia, adaptando colores y formas, acercando la identidad de los manuales a otros programas implementados por el Gobierno Federal, como #Tamojunto y el Programa Elos^12^. También se revisaron los materiales, proponiendo cambios en algunas actividades escritas que comenzaron a realizarse en formato oral, dada la demanda de los participantes con bajo nivel de alfabetización, dada la sobrecarga de facilitadores en la preparación de los materiales y la exigencia de los participantes de llevarse el material a casa. Así, el material permanente (tarjetas y pósteres) y los cuadernos de actividades para padres y jóvenes se crearon en formato de kit. Incluso ante las adaptaciones, el temario y el número de reuniones no cambiaron en los elementos centrales del programa.

La variable de resultado “consumo de alcohol en adolescentes” se analizó mediante preguntas extraídas del cuestionario de la Organización Mundial de la Salud (OMS), utilizado por el Centro Brasileño de Información sobre Drogas Psicotrópicas (CEBRID), en la VI Encuesta sobre el Consumo de Drogas entre Estudiantes^12^. Para este estudio, se utilizaron las siguientes preguntas para analizar el consumo de alcohol (sí o no) y la práctica del consumo excesivo de alcohol en la vida (sí o no): “¿Has probado alguna vez alguna bebida alcohólica?” y “¿Has tomado alguna vez 5 dosis o más de alcohol en la misma ocasión?”.

En relación con el “consumo de alcohol y otras drogas por adultos”, se aplicó el instrumento *Alcohol Use Disorders Identification Test* (AUDIT) (“Prueba de Identificación de Trastornos por Consumo de Alcohol”), que examina a las personas en riesgo de desarrollar trastornos relacionados con el alcohol^13^. La versión brasileña completa del AUDIT^14^, que incluye 10 elementos, cubre tres áreas principales: consumo de alcohol (puntos 1 a 3), dependencia al alcohol (puntos 4 a 6) y consecuencias negativas (puntos 7 a 10). La puntuación total, que va de cero a 40 puntos, suele calcularse y utilizarse para evaluar el riesgo asociado al consumo de alcohol. Esta puntuación permite clasificar a los participantes en diferentes categorías, como consumo de alcohol de bajo riesgo o abstinente (0 a 7 puntos), riesgo de uso peligroso (8 a 15 puntos), consumo dañino de alcohol (16 a 19 puntos) o dependencia del alcohol (20 a 40 puntos)^15^.

La variable sobre las creencias parentales se midió mediante cinco preguntas con respuestas dicotómicas (sí o no): a) “Creo que es más seguro enseñar a mi hijo a beber en casa mientras es adolescente”, b) “Si mi hijo no bebe, será excluido del grupo de amigos”, c) “Emborracharse en la adolescencia es solo una fase y pasará pronto”. d) “Si un adolescente solo bebe en fiestas, no afectará su desarrollo”, e) “El alcohol no es una droga, es comida”.

Los datos sociodemográficos incluían género, educación, edad y estatus socioeconómico, estos últimos recopilados mediante la escala de la Asociación Brasileña de Empresas de Investigación (ABEP)^16^. Las puntuaciones más altas indican una mejor posición económica, y las clases socioeconómicas se clasifican de la A (la más alta) a la E (la más baja), y la puntuación se genera a partir de la información sobre bienes de consumo y la escolarización del cabeza de familia. Según el ABEP, las clases D/E pueden agruparse. Todas las variables utilizadas en este estudio se recogieron en la línea inicial y, por tanto, no fueron aleatorizadas ni influenciadas por la intervención.

Los grupos de control e intervención fueron sometidos al mismo cuestionario virtual y guiado por audio en tres momentos: antes del inicio de la intervención, seis meses después y 12 meses después. El cuestionario estuvo disponible para los participantes a través de *smartphones*, por investigadores vinculados a las universidades que promovían el estudio, sin la presencia del facilitador del programa. La finalización fue anónima y se aplicó el mismo cuestionario en los tres momentos de recogida de datos; Sin embargo, a los cuestionarios de seguimiento se añadirán algunas preguntas sobre la experiencia de participar en el programa. El cuestionario fue previamente completado por los investigadores de campo con un código de municipio y CRAS. Este código permitía el análisis por clústeres (multinivel) en dos niveles (municipio y servicio). Destacan las experiencias previas del equipo con esta plantilla de código sigiloso de emparejamiento temporal. Se añadió un código familiar y un código CRAS al código individual, para permitir el emparejamiento entre adolescentes y la identificación del clúster de CRAS.

Los datos se recogieron utilizando cuestionarios estructurados, virtuales y anónimos con autocompletación planificada, basados en referencias internacionales utilizadas para la evaluación SFP 10-14^12^. La aplicación del instrumento se realizó en un lugar privado dentro del CRAS o en una estructura indicada por el servicio. Los investigadores pusieron a disposición tabletas o ordenadores para que las familias los rellenaran. Al rellenarlo, padres y adolescentes estaban en un lugar separado, para preservar la confidencialidad, el secretismo y, especialmente, por parte de los participantes adolescentes, evitando la sensación de que los datos se socializarían con los padres.

Las encuestas con muchos sujetos y seguimiento temporal tienden a perder el seguimiento. Teniendo en cuenta el tamaño del ensayo controlado presentado, se adoptó la imputación múltiple de datos para reducir el sesgo y aumentar la fiabilidad de los resultados. A lo largo de la investigación, se adoptaron técnicas motivacionales y conductuales con las familias, especialmente con los adolescentes, estimulando la participación en el programa y la realización de cuestionarios.

Teniendo en cuenta la sensibilidad de los temas tratados, se adoptaron algunas iniciativas para asegurar una reducción del sesgo informativo; los procedimientos de campo fueron: 1) anunciar a los sujetos que habían recibido un cuestionario sobre salud y comportamientos; 2) uno o dos investigadores formados estuvieron presentes durante el proceso de relleno de los cuestionarios y no se permitía la presencia de ningún profesional del CRAS en ese momento de la recogida; 3) los cuestionarios se rellenaron electrónicamente en *smartphones* individuales para evitar el contacto entre los investigadores y los cuestionarios completados, asegurando así una mayor confidencialidad de la información; 4) los investigadores de campo demostraron más de una vez que el cuestionario es anónimo y que los datos no han sido ni serán consultados por nadie más que los propios investigadores; 5) los jóvenes respondían a los cuestionarios en salas separadas, acompañados por un miembro del equipo de investigación.

### Análisis estadístico

Las variables estudiadas se describieron inicialmente según la naturaleza de las medidas y se resumieron mediante números absolutos y prevalencia. En el segundo momento, se realizó una prueba de hipótesis para evaluar la asociación entre las variables de interés y el consumo de alcohol por parte de los adolescentes utilizando la prueba de chi-cuadrado. Posteriormente, se consideró el modelo inicial de regresión logística multivariante, incluyendo las variables explicativas que presentaron un valor de p ≤ 0,20 en la regresión univariante. A continuación, se utilizó un procedimiento retroactivo para eliminar las variables explicativas con p > 0,05, con el fin de obtener un modelo final para cada variable de respuesta, las respectivas *odds ratios* (OR), el intervalo de confianza (IC) del 95% y los valores p. El nivel de significación adoptado fue del 5%.

Los análisis se realizaron en ambas muestras: “intención de tratar” (ITT), incluyendo a todos los sujetos que habían sido aleatorizados para participar en el estudio, según el grupo de asignación original, independientemente de si habían perdido los datos; así como los “Casos Completos” (CC), en los que solo se consideraban observaciones con datos completos en el momento inicial y seguimientos; por lo tanto, en esta muestra, se excluyeron los casos con valores faltantes.

Las medidas de asociación se analizaron utilizando *Generalized Linear Mixed Models* (“Modelos Lineales Mixtos Generalizados”) (GLMM), que calculan el nivel de dependencia entre los datos. Al considerar la estructura jerárquica de los datos, se utilizaron modelos multinivel para verificar simultáneamente diferencias en la prevalencia de consumo entre tiempos y entre grupos, ponderando las escalas como el nivel de modelación^12^. Para la construcción, organización y presentación metodológica, el estudio está alineado con el *checklist Strengthening the Reporting of Observational Studies in Epidemiology* (STROBE).

## Resultados

La Tabla 1 muestra que la edad media de los adolescentes era de 12,6 años y, para los padres, de 39,5 años, la mayoría de los cuales pertenecían al estatus socioeconómico de la categoría D/E (73,2%). En cuanto al consumo de drogas en la vida, el 12,3% de los adolescentes declaró haber consumido alcohol y el 3,6% tenía consumo excesivo de alcohol. En cuanto a los padres, la mayoría (96,3%) tenía bajo riesgo de consumo de alcohol. En cuanto a las creencias parentales sobre el consumo de alcohol por parte de los adolescentes, se destaca que el 10,5% cree que es más seguro enseñar a sus hijos a beber en casa y el 20,8% afirma que los adolescentes que solo beben en fiestas no verán afectado su desarrollo.

**Tabla 1:** Características sociodemográficas de los adolescentes y adultos participantes en el estudio (n = 859). Fortaleza, CE, Brasil, 2021

Variables	Adolescentes		Adultos	
	(n= 884)		(n= 884)	
	n*	%^†^	n*	%^†^
**Sexo**				
Masculino	414	48,2	72	8,4
Femenino	439	51,1	782	91,0
Otros	6	0,7	5	0,6
**Edad (media DE)** ^‡^	12,6±1,24		39,50±8,15	
**Estatus socioeconómico**				
A	---	---	8	0,9
B	---	---	33	3,8
C	---	---	190	22,1
D/E	---	---	628	73,2
**Consumo de drogas en la vida (adolescentes)**				
Alcohol	104	12,3	---	---
Consumo excesivo de alcohol	30	3,6	---	---
Consumo de alcohol por parte de los responsables				
Bajo riesgo o abstenentes	---	---	827	96,3
Riesgo	---	---	21	2,4
Uso dañino o probable dependencia	---	---	11	1,3
**Creencias sobre el consumo de drogas**				
Consumo seguro en casa^§^	---	---	89	10,5
Ser excluido^||^	---	---	149	17,5
Borracho en la adolescencia^¶^	---	---	116	13,7
Beber en fiestas**	---	---	177	20,8
El alcohol no es una droga^††^	---	---	45	5,3

*n = Tamaño de la muestra; ^†^Valor porcentual; ^‡^Valor medio de edad; ^§^Creo que es más seguro enseñar a mi hijo a beber en casa mientras es adolescente; ^||^Si mi hijo no beber, será excluido del grupo de amigos; ^¶^Emborracharse en la adolescencia es solo una fase y pronto pasará; **Si un adolescente solo beber en fiestas, no afectará su desarrollo; ^††^Alcohol no es droga, es comida

La Tabla 2 muestra que el consumo de alcohol, así como el consumo excesivo de alcohol, es más prevalente en adolescentes varones. Del mismo modo, la edad es un parámetro relevante y es evidente que los adolescentes de 13 y 14 años consumen más en comparación con los de 11 y 12 años; Esto quedó demostrado estadísticamente. En el análisis de creencias parentales, entre los adolescentes que consumieron bebidas alcohólicas, el 19,3% tenía padres que creían que era más seguro enseñar a su hijo a beber en casa mientras era adolescente (p=0,038). Por otro lado, las creencias de que “emborracharse en la adolescencia es solo una fase y pasará pronto” y “si un adolescente solo bebe en fiestas, no afectará su desarrollo” se asociaron con la práctica del consumo excesivo de alcohol por parte de los adolescentes (p=0,037 y p=0,010, respectivamente).

**Tabla 2:** Distribución del consumo y consumo excesivo de alcohol entre adolescentes, según variables sociodemográficas, creencias parentales y consumo de alcohol por parte de los padres. Fortaleza, CE, Brasil, 2021

	Consumo de alcohol en la vida					Consumo excesivo de alcohol en la vida				
Variables	No		Sí		P^‡^	No		Sí		P^‡^
	n*	%^†^	n*	%^†^		n*	%^†^	n^*^	%^†^	
**Sexo del adolescente**										
Masculino	352	47,3	58	55,8	0,238	382	47,3	19	63,3	0,212
Femenino	388	52,1	45	43,3		420	52,0	11	36,7	
Otros	5	0,6	1	0,9		6	0,7	0	-	
**Edad del adolescente**										
10-12 años	365	49	20	19,2	<0,001	370	45,8	9	30	0,088
13-14 años	380	51	84	80,8		438	54,2	21	70	
**Asociación Brasileña de Empresas de Investigación**										
A	7	0,9	0	-	0,684	8	1	0	-	0,813
B	28	3,8	5	4,8		31	3,8	1	3,3	
C	163	21,9	25	24		181	22,4	5	16,7	
D/E	547	73,4	74	71,2		588	72,8	24	80	
**Creencias de los padres**										
Consumo seguro en casa^§^	71	80,7	17	19,3	**0,038**	84	97,7	2	2,3	0,603
Ser excluido^||^	129	87,2	19	12,8	0,771	141	95,9	6	4,1	0,669
Borracho en la adolescencia^¶^	97	83,6	19	16,4	0,159	106	93	8	7,0	**0,037**
Beber en fiestas**	152	86,9	23	13,1	0,716	163	93,1	12	6,9	**0,010**
El alcohol no es una droga^††^	37	86,1	6	13,9	0,741	42	95,5	2	4,5	0,731
**Consumo de alcohol por parte de los padres**										
Consumo de bajo riesgo o abstenentes	720	88,1	97	11,9	**<0,001**	778	96,4	29	3,6	0,769
Consumo de riesgo	20	95,2	1	4,8		19	95	1	5,0	
Uso dañino o probable dependencia	5	45,5	6	54,5		11	100	0	-	

*n = Tamaño de la muestra; ^†^Valor porcentual; ^‡^p = valor p en el nivel de 5% (p ≤ 0,05); ^§^Creo que es más seguro enseñar a mi hijo a beber en casa mientras es adolescente; ^||^Si mi hijo no beber, será excluido del grupo de amigos; ^¶^Emborracharse en la adolescencia es solo una fase y pronto pasará; **Si un adolescente solo beber en fiestas, no afectará su desarrollo; ^††^El alcohol no es una droga, es comida

La Tabla 3 destaca la asociación entre el consumo de alcohol de los padres y las creencias sobre el consumo de alcohol y el consumo excesivo de alcohol por parte de sus hijos. Se observó que los padres que “creen que es más seguro enseñar a sus hijos a beber en casa” duplicaban las probabilidades de que sus hijos empezaran a consumir alcohol (OR = 2,10). En relación con los padres que afirmaron creer que “si un adolescente solo bebe en fiestas, no afectará su desarrollo”, las probabilidades de que sus hijos beban en exceso casi se triplicaron (OR= 2,61). Otro hallazgo importante fue que los padres o tutores que hicieron uso perjudicial o tenían probable dependencia del alcohol aumentaron las probabilidades de consumo de alcohol por parte de sus hijos en ocho veces (OR= 8,91).

**Tabla 3:** Asociación entre el inicio del consumo de alcohol, el consumo excesivo de alcohol (adolescentes) y las creencias parentales y el consumo de alcohol por parte de los padres, según estimación en el OR* y el intervalo de confianza del 95%^†^ (N = 854). Fortaleza, CE, Brasil 2021

	Regresión Logística Univariada						Regresión Logística Multivariada					
	Consumo de alcohol en la vida			Consumo excesivo de alcohol			Consumo de alcohol en la vida			Consumo excesivo de alcohol		
Variables	OR*	95%IC^†^	p^‡^	OR*	95%IC^†^	p^‡^	OR*	95%IC^†^	p^‡^	OR*	95%IC^†^	p^‡^
**Creencia de los padres**												
Consumo seguro en casa^§^	1,82	[1,03;3,24]	0,041	0,68	[0,16;2,93]	0,605	2,10	[1,04;3,53]	0,036	---	---	--
Ser excluido^||^	1,08	[0,63;1,85]	0,771	1,22	[0,49;3,05]	0,669	---	---	--	---	---	--
Borracho en la adolescencia^¶^	1,47	[0,86;2,53]	0,161	2,37	[1,03;5,47]	0,042	---	---	---	---	---	--
Beber en fiestas**	1,10	[0,67;1,80]	0,716	2,61	[1,23;5,53]	0,012	---	---	---	2,61	[1,23;5,53]	0,012
Alcohol no es una droga^††^	1,16	[0,48;2,82]	0,741	1,29	[0,30;5,61]	0,732	---	---	--	---	---	--
**Consumo de alcohol por parte de los padres**												
Consumo de bajo riesgo o abstenentes	ref			ref			ref			ref		
Consumo de riesgo	0,37	[0,05; 2,80]	0,336	1,41	[0,18; 10,91]	0.741	---	---	---	---	---	---
Uso dañino o probable dependencia	8,91	[0,67; 29,74]	<0.001	---	---	---	6,41	[1,84;22,34]	0,004	---	---	---

^*^OR *= Odds Ratio*; ^†^Intervalo de Confianza de 95%; ^‡^p = valor p en el nivel de 5% (p ≤ 0,05); ^§^Creo que es más seguro enseñar a mi hijo a beber en casa mientras es adolescente; ^||^Si mi hijo no beber, será excluido del grupo de amigos; ^¶^Emborracharse en la adolescencia es solo una fase y pronto pasará; **Si un adolescente solo beber en fiestas, no afectará su desarrollo; ^††^El alcohol no es una droga, es comida

## Discusión

El estudio identificó que algunas creencias, como enseñar al niño a beber en casa y pensar que beber en fiestas no es un problema, están asociadas con el inicio del consumo de alcohol a una edad temprana y la práctica del consumo excesivo de alcohol en la adolescencia. Además, se identificó que los padres que presentaban un consumo nocivo de alcohol o que tenían probable dependencia aumentaban hasta ocho veces las probabilidades de que los adolescentes iniciaran el consumo de bebidas alcohólicas, en comparación con quienes no consumían bebidas nocivas. La relevancia de estos datos reitera la necesidad de que las enfermeras trabajen con padres y adolescentes sobre las creencias.

Los adolescentes en este estudio tenían una edad media de 12,6 años y consumo en aproximadamente el 12,3% de los participantes. Esta realidad es muy similar al contexto nacional, ya que la media ronda los 13 años^17^. En comparación con países con perfiles socioeconómicos similares, como México y Tailandia, es aún más común que los adolescentes experimenten con el alcohol a una edad temprana, lo que puede alcanzar una prevalencia del 18% al 60% en este mismo grupo de edad^18^.

En cuanto a los adultos que consumen alcohol, en este estudio, el 5,8% hizo un uso perjudicial o probablemente dependiente del alcohol, pero cabe destacar que, en esta muestra, el 91% de los adultos eran mujeres. Socialmente, las mujeres son más vulnerables y, en el consumo de alcohol, esto revela un escenario preocupante con consecuencias, de forma contundente, en la salud mental. A pesar de ello, el escenario brasileño se caracteriza de forma opuesta, ya que se percibe que, entre los bebedores empedernidos, la prevalencia fue del 3,2% entre los hombres y del 0,6% entre las mujeres^19^.

El presente estudio encontró una fuerte asociación entre el consumo de alcohol por parte de los padres y el consumo de alcohol por adolescentes. Se identificó que los padres o tutores que consumían alcohol o tenían una probable dependencia aumentaba las probabilidades de consumo de alcohol por parte de los adolescentes en más de ocho. De manera similar, un estudio en la Región Sur de Brasil, con 1.170 adolescentes, mostró que el 48% de los que consumieron informaron que sus padres consumían bebidas alcohólicas y que el 10,5% de estos padres tuvieron algún problema con el alcohol en los últimos 30 días^20^. Otro estudio en Brasil mostró que el consumo de bebidas alcohólicas en casos familiares de consumo por parte de madres y padres se asoció con el consumo excesivo de alcohol en adolescentes^21^. Esta correlación en el consumo de los jóvenes puede explicarse por la relación parental y el ejemplo evidenciado en la familia; Así, los jóvenes reflejan su comportamiento en el modelo de crianza al que están sometidos^22^.

En cuanto a las creencias parentales sobre el consumo de alcohol por parte de sus hijos, se encontró que los adultos que respondieron que consideraban más seguro enseñar a sus hijos a beber en casa aumentaron considerablemente las probabilidades de que sus hijos empezaran a beber temprano, con una proporción de 1,97 más de probabilidades de comenzar este consumo. Además, el 20,8% de los adultos cree que, si los adolescentes solo beben en fiestas, esta práctica no afectará a su desarrollo. Esta creencia, en el contexto del estudio, puede casi duplicar las probabilidades de que los niños beban en exceso. Se señaló la práctica de consumir en fiestas como un desencadenante para el consumo excesivo, además de que el ambiente de fiesta se asociaba con el lugar donde se consumió por primera vez alcohol u otras drogas. La práctica del consumo excesivo de alcohol influye negativamente en los procesos de aprendizaje de los adolescentes, predisponiéndolos a la adicción a las drogas, al consumo de cigarrillos y drogas ilícitas, así como a crisis de abstinencia^23^.

En familias cuyos padres consumen sustancias psicoactivas, existe una mayor probabilidad de consumo entre sus hijos. Se presume que la asociación significativa entre la práctica de los padres/tutores que beben y el policonsumo entre los estudiantes puede reflejar la influencia parental en los adolescentes^24^. El entorno familiar es el principal grupo social de adolescentes durante el proceso de crecimiento y puede actuar como un factor desencadenante para el consumo de sustancias psicoactivas. La relación entre el comportamiento del joven y el de los padres puede estar asociada con la reproducción de patrones observados en la vida familiar.

Por otro lado, los padres o tutores que respondieron positivamente que “si un adolescente solo bebe en fiestas, no afectará a su desarrollo” resultaron en un aumento de 2,61 veces en las probabilidades de que los adolescentes inicien el consumo de alcohol. Mientras que quienes respondieron “Emborracharse en la adolescencia es solo una fase y pronto pasará”, hubo un aumento de 2,37 veces en la probabilidad de consumo de alcohol. De forma similar a los hallazgos, en una revisión sistemática se señaló que las actitudes parentales menos restrictivas hacia el consumo de alcohol entre los jóvenes se asociaron con tasas más altas de inicio del consumo de alcohol (OR) = 1,45, frecuencia de consumo (OR = 1,52) y episodios de embriaguez (OR = 1,58)^25^.

El comportamiento parental en relación con el consumo de alcohol está vinculado a factores como el entorno familiar, el estilo de crianza, los límites y las influencias culturales. Los padres son conscientes de los riesgos del alcohol y desaprueban su consumo por parte de los jóvenes, pero reconocen su presencia inevitable en la cultura. Así, muchos adoptan una postura equilibrada y receptiva a regañadientes, buscando reconciliar los valores personales con la promoción de la autonomía de sus hijos y su adaptación a las normas sociales^26^.

El programa mostró un aumento significativo en la capacidad de los padres para establecer normas claras sobre el consumo de drogas de sus hijos tras 24 meses de seguimiento. La literatura sobre prevención del consumo de sustancias muestra de forma consistente que las normas parentales bien definidas y firmes son un factor protector importante contra el inicio y la progresión del consumo entre los adolescentes. Estas normas ayudan a crear límites objetivos, permitiendo a los jóvenes comprender las expectativas familiares y sociales respecto al consumo de drogas, además de reforzar normas de comportamiento que desincentivan este consumo. En este contexto, los efectos del programa en la reducción de la negligencia parental y el fortalecimiento de las prácticas normativas pueden tener un impacto considerable en la prevención del consumo de sustancias. Investigaciones futuras deberían investigar, mediante modelos de mediación, si estos cambios positivos en los comportamientos parentales, junto con la reducción de la negligencia, influyen directamente en la prevención del consumo de drogas entre los adolescentes^27^.

Cabe señalar que la permisividad de los padres respecto al consumo de alcohol por parte de los adolescentes está asociada a un daño a largo plazo, independientemente de la edad a la que comenzó el consumo^28^. En vista de esto, las enfermeras asumen un papel estratégico en la prevención, actuando en la identificación y deconstrucción de creencias que normalizan el consumo temprano de alcohol. Es fundamental que las enfermeras desarrollen acciones educativas planificadas, especialmente en el entorno escolar, con el objetivo de sensibilizar a los padres, estudiantes y a la comunidad sobre los riesgos implicados. Estas intervenciones contribuyen a la reducción de factores de riesgo y a la promoción de entornos más protectores para los adolescentes.

Según lo anterior, al identificar el perfil de consumo entre los adolescentes, es importante considerar que el consumo de alcohol puede favorecer conductas violentas, peleas, causar desinhibición y hacer que los adolescentes sean más vulnerables^29^. Por lo tanto, conocer el contexto de las creencias parentales es relevante para la atención sanitaria, principalmente porque cada estilo parental tiene implicaciones significativas para el desarrollo de los niños y puede influir en el bienestar emocional, social y cognitivo a lo largo de la vida.

Se ha demostrado que las intervenciones dirigidas a padres que refuerzan la monitorización, la comunicación eficaz y el apoyo emocional son efectivas. Estas estrategias reducen directamente la disponibilidad y asequibilidad del alcohol para los adolescentes. Además, actúan de forma indirecta, influyendo en comportamientos y percepciones adultas, promoviendo entornos regulatorios más estrictos y normas parentales más saludables, ambos factores asociados con la reducción del consumo de alcohol por menores^30^ ^-^ ^31^.

Es en este contexto que programas de prevención basados en la evidencia, como el Programa de Familias Fuertes, propuesto por el Gobierno Federal brasileño, proponen intervenciones que actúan sobre la calidad de las relaciones familiares, desarrollando un apego seguro entre niños y padres, incluyendo disciplina afectiva, supervisión y supervisión parental. El desarrollo de estas habilidades se realiza de forma programada, planificada y sistemática, favoreciendo a las familias para aprender y entrenar estas habilidades de forma interactiva, siendo la forma de aprendizaje más eficaz^32^.

Las evidencias indican que los padres tienden a reportar niveles más altos de supervisión parental que sus hijos, y que estas discrepancias son mayores en comportamientos de establecimiento de normas que en las solicitudes parentales. Además, con el tiempo, tanto padres como jóvenes informan de una disminución en las normas parentales, aunque solo los padres informan de una reducción en las solicitudes. En vista de esto, es esencial examinar la percepción de los jóvenes sobre la supervisión parental en relación con el consumo de alcohol, especialmente entre adolescentes con diferentes patrones de consumo (es decir, abstemios y consumidores), especialmente cuando alcanzan la edad mínima legal para beber. Además, se ha demostrado que normas específicas sobre el consumo de alcohol son efectivas para reducir el consumo en estudios de intervención^33^ ^)-(^ ^34^.

Entre las limitaciones del estudio destaca el hecho de que se realizara solo en doce ciudades brasileñas, lo que requiere precaución al generalizar los resultados a la población brasileña, debido a la amplia diversidad geográfica y cultural. Además, los cuestionarios se aplicaron en un entorno de red de apoyo social, lo que pudo haber influido en las respuestas, debido a la presencia de colegas y al contexto social. Aunque la recogida de datos se realizó por separado de sus padres, en ciertas etapas es posible que los adolescentes sintieran algún nivel de juicio o recriminación, aunque solo fuera en su imaginación.

Los resultados ofrecen un apoyo importante para los profesionales que trabajan en el área de la salud adolescente, ya que señalan las creencias parentales como un problema relevante de salud pública. Por ello, es esencial promover intervenciones tempranas destinadas a prevenir conductas de riesgo. En el contexto brasileño, la cultura permisiva en relación con el consumo de alcohol en la adolescencia, combinada con el frágil control sobre la venta de bebidas alcohólicas, refuerza la urgente necesidad de invertir en investigación que profundice la comprensión de estas vulnerabilidades.

## Conclusión

El estudio señaló que las creencias parentales permisivas, como el consumo de bebidas alcohólicas en casa y en fiestas y el uso nocivo del alcohol por parte de los padres, pueden aumentar considerablemente las probabilidades de que los niños inicien temprano el consumo de alcohol y la práctica del consumo excesivo de alcohol. Por tanto, el consumo de bebidas alcohólicas por parte de los padres es un factor agravante para la salud de los adolescentes, provocando pérdidas en las prácticas de vida.

Por tanto, es importante desarrollar programas de prevención para familias cuyos miembros aún están en la fase temprana de la adolescencia, y que no solo aclare las consecuencias del consumo temprano de bebidas alcohólicas, sino también trabajar en prácticas de educación parental, modificando las creencias parentales consideradas en riesgo de consumo de alcohol en la adolescencia, implicando prevención. Finalmente, se destacan las diversas posibilidades de acciones para que las enfermeras trabajen los factores protectores, incluyendo creencias sobre el consumo de alcohol y el consumo excesivo de alcohol con padres y adolescentes, en el ámbito de la prevención. Además, los estudios que evalúan las creencias de los padres y su conocimiento sobre el daño que el alcohol puede causar en la vida de los adolescentes son importantes para guiar el desarrollo de estos programas.

## Data Availability

Los conjuntos de datos relacionados con este artículo estarán disponibles previa solicitud al autor correspondiente.
